# *In Situ* Cellular Localization of Nonfluorescent [60]Fullerene Nanomaterial in MCF-7 Breast Cancer Cells

**DOI:** 10.1021/acsbiomaterials.2c00542

**Published:** 2022-07-20

**Authors:** Maciej Serda, Katarzyna Malarz, Julia Korzuch, Magdalena Szubka, Maciej Zubko, Robert Musioł

**Affiliations:** † Institute of Chemistry, University of Silesia in Katowice, Katowice, 40-006, Poland; ‡ Silesian Center for Education and Interdisciplinary Research, 75 Pulku Piechoty 1a, 41-500 Chorzow, Poland; § Chełkowski Institute of Physics, University of Silesia in Katowice, 75 Pulku Piechoty 1, 41-500 Chorzow, Poland; ∥ Institute of Materials Engineering, University of Silesia in Katowice, 75 Pulku Piechoty 1A, 41-500 Chorzow, Poland; ⊥ Department of Physics, Faculty of Science, University of Hradec Králové, Rokitanského 62, 500 03 Hradec Králové, Czech Republic

**Keywords:** [60]fullerenes, click reactions, cellular colocalization, breast cancer, lysosomes, triazoles

## Abstract

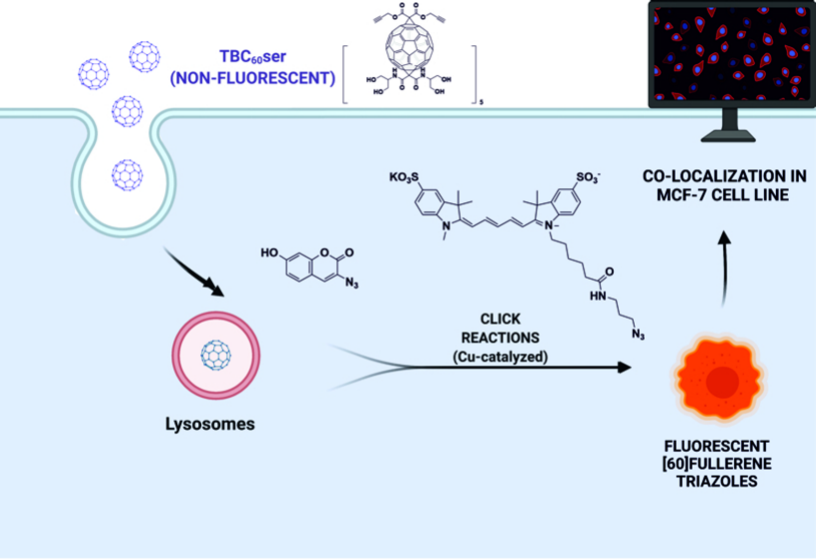

Cellular localization of carbon nanomaterials in cancer cells is essential information for better understanding their interaction with biological targets and a crucial factor for further evaluating their biological properties as nanovehicles or nanotherapeutics. Recently, increasing efforts to develop promising fullerene nanotherapeutics for cancer nanotechnology have been made. However, the main challenge regarding studying their cellular effects is the lack of effective methods for their visualization and determining their cellular fate due to the limited fluorescence of buckyball scaffolds. Herein, we developed a method for cellular localization of nonfluorescent and water-soluble fullerene nanomaterials using the *in vitro* click chemistry approach. First, we synthesized a triple-bonded fullerene probe (TBC_60_ser), which was further used as a starting material for 1,3-dipolar cycloaddition using 3-azido-7-hydroxycoumarin and sulfo-cyanine5 azide fluorophores to create fluorescent fullerene triazoles. In this work, we characterized the structurally triple-bonded [60]fullerene derivative and confirmed its high symmetry (*T*_*h*_) and the successful formation of fullerene triazoles by spectroscopic techniques (i.e., ultraviolet–visible, fluorescence, and Fourier transform infrared spectroscopies) and mass spectrometry. The created fluorescent fullerene triazoles were successfully localized in the MCF-7 breast cancer cell line using fluorescent microscopy. Overall, our findings demonstrate that TBC_60_ser localizes in the lysosomes of MCF-7 cells, with only a small affinity to mitochondria.

## Introduction

At present, nanomedicine is entering clinical trials, and some nanotherapeutics have already been approved by the FDA and EMA to treat several lethal diseases, including cancer and microbial infections.^1,2^ Compared with traditional treatment modalities, engineered nanoparticles offer new therapeutic options, especially for breast cancer, where nanotherapeutics such as Doxil and Abraxane have already been used in adjuvant therapies.^3^ Breast tumors are heterogeneous and complex pathogenic entities, and those that do not express crucial hormone receptors (e.g., triple-negative breast cancer) are significantly more invasive and apt to metastasize.^4^ Carbon nanomaterials have attracted significant interest in cancer nanotechnology, especially as drug delivery vehicles and theranostic pharmaceuticals on a nanometric scale.^5−7^ Some examples of nanotherapeutics include a multifunctional drug delivery system with transferrin/hyaluronic acid-functionalized multiwalled carbon nanotubes (HA-MWCNTs/Tf@ART) for *in vitro* treatment of breast cancer cells as well as [60]fullerene nanoconjugate with docetaxel, which significantly improve its bioavailability.^8,9^

Biological uptake and cellular and organ localization of engineered nanoparticles are crucial information when developing novel carbon nanomaterials for cancer nanotechnology and beyond.^10^ Several analytical techniques have been developed to study the localization of nanomaterials in biological samples, including fluorescent, intravital, and transmission electron microscopies.^11,12^ These techniques have been used to study biodistribution and uptake/clearance of carbon nanomaterials in cancer tissues in living organisms and traditional two-dimensional cellular cultures, especially for fluorescent carbon dots, near-infrared absorbing carbon nanotubes, and fluophore-labeled fullerenes.^13−15^ Fullerene derivatives have been studied extensively in the last 30 years, as there are several synthetic approaches to make them water-soluble. This includes reactions with strong bases in the presence of quaternary ammonium salts, interactions with polyhydroxylated sugars, as well as Bingel–Hirsch/Prato reactions with substrates possessing a large number of amine, hydroxyl, or carboxylic functional groups.^16,17^ After the formation of fully water-soluble [60]fullerene nanomaterials, they were intensively explored in terms of their cytotoxicity profiles and cell uptake models.^18,19^ Nevertheless, detection of fullerene derivatives in cells is limited due to their drastically weak fluorescence in polar solvents.^20^ Some of the literature methods reported to overcome this inconvenience rely on nonspecific complexation with fluorophores, synthesizing fluorescently labeled fullerene derivatives, or visualization of fullerene nanomaterials using a special fullerene antibody.^19,21^ Another approach was proposed by Di Giosia and co-workers, who synthesized a water-soluble C_70_@lysozyme complex and confirmed its localization in lysosomes using photoacoustic and third-harmonic generation (THG) imaging techniques.^22^ Most of the above-mentioned methods share an important drawback related to substantial changes in the properties of the nanomaterial. Particularly, covalent binding of the fluorophore to the buckyball may drastically alter not only physicochemical parameters but also essentially the affinity of the latter toward biological targets and its fate in the cellular environment. Fullerene-specific antibodies are apparently free from these drawbacks but introduce others related to the chemical nature of the monoclonal antibody as well as unacceptable specificity at times, especially to fullerene derivatives.

The development of “click chemistry” changed the fields of organic synthesis and nanotechnology, opening novel possibilities for drug development and bioconjugation;^23,24^ its methodology is mainly based on copper(I)-catalyzed 1,3-dipolar cycloadditions between organic azides and alkynes, resulting in 1,4-disubstituted 1,2,3-triazoles as variously functionalized molecular scaffolds. These click reactions have been successfully used to efficiently functionalize engineered carbon nanomaterials, including CNTs, graphene oxide, and fullerenes.^25−27^ Classical works by groups led by Nierengarten and Martin described the formation of very complex fullerene nanomaterials using various copper catalysts (e.g., CuSO_4_·5H_2_O and sodium ascorbate), often with fascinating supramolecular and biological properties, such as Ebola virus inhibition or liquid crystal formation.^28,29^ However, to the best of our knowledge, there are no reports describing the use of *in situ* copper(I)-catalyzed click reactions in cancer cells to confirm the cellular localization of nonfluorescent fullerene nanomaterials. Our observation is of great practical importance to all cancer nanotechnology scientists working with water-soluble fullerenes and studying their biodistribution. Owing to previous works on concentration-dependent cytotoxicity of copper(I) salts in cellular conditions,^30,31^ novel synthetic approaches were developed. These include famous works by Bertozzi and co-workers describing “biorthogonal reactions”, which could be performed even in living organisms (including humans) using a plethora of cyclooctyne derivatives and appropriate organic azides in copper-free conditions.^32^ Moreover, novel cyclooctyne derivatives of [60]fullerene were also created for bioorthogonal reactions, but they are not soluble in water, and no reports have been published describing their direct translation for *in vivo* experiments.^33^

**Figure 1:**
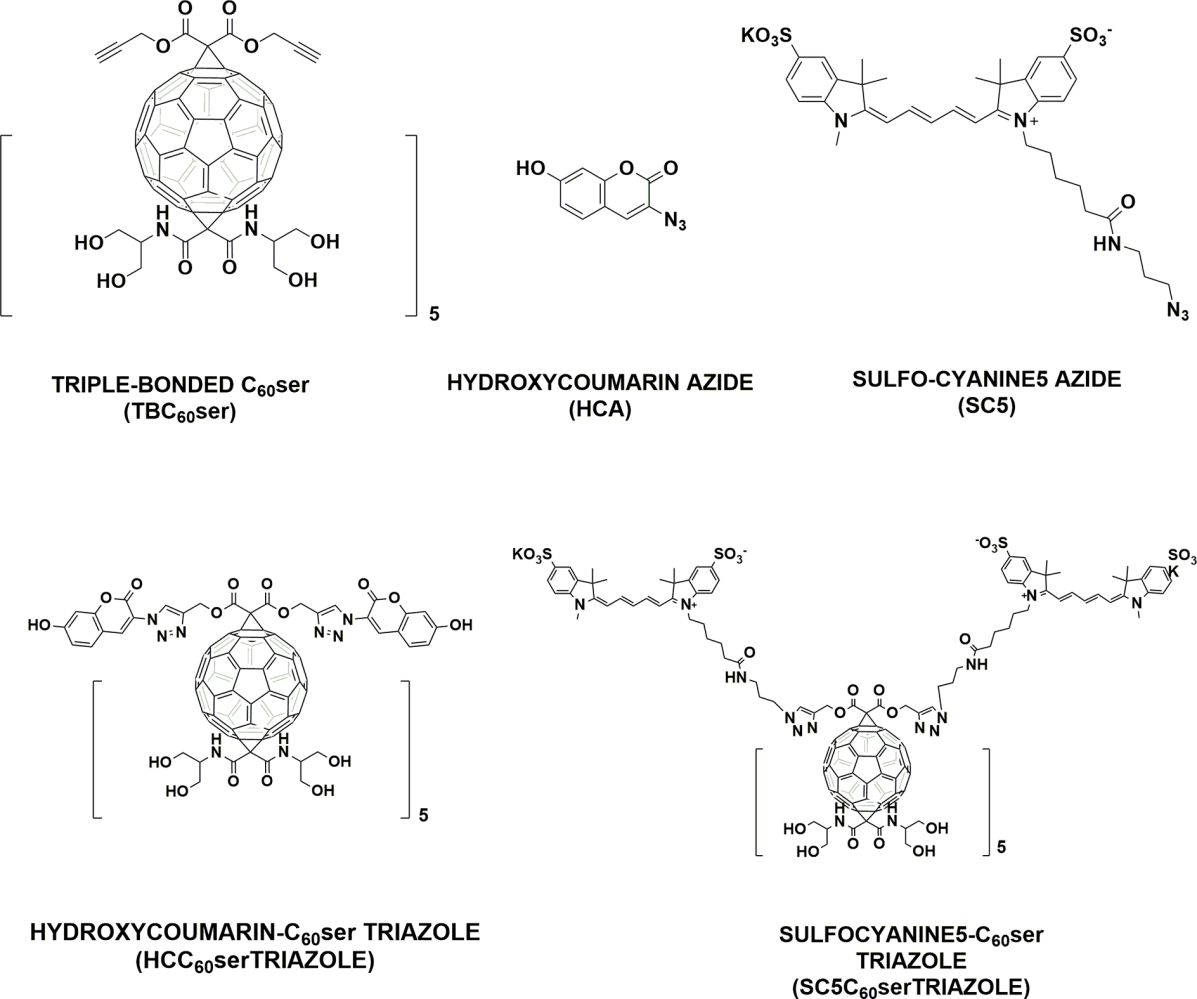
Chemical structures of triple-bonded[60]fullerene nanomaterial C_60_ser (TBC_60_ser) and fluorescent probes (HCA and SC5) that were used for its visualization and formation of fullerene triazoles.

**Scheme 1:**
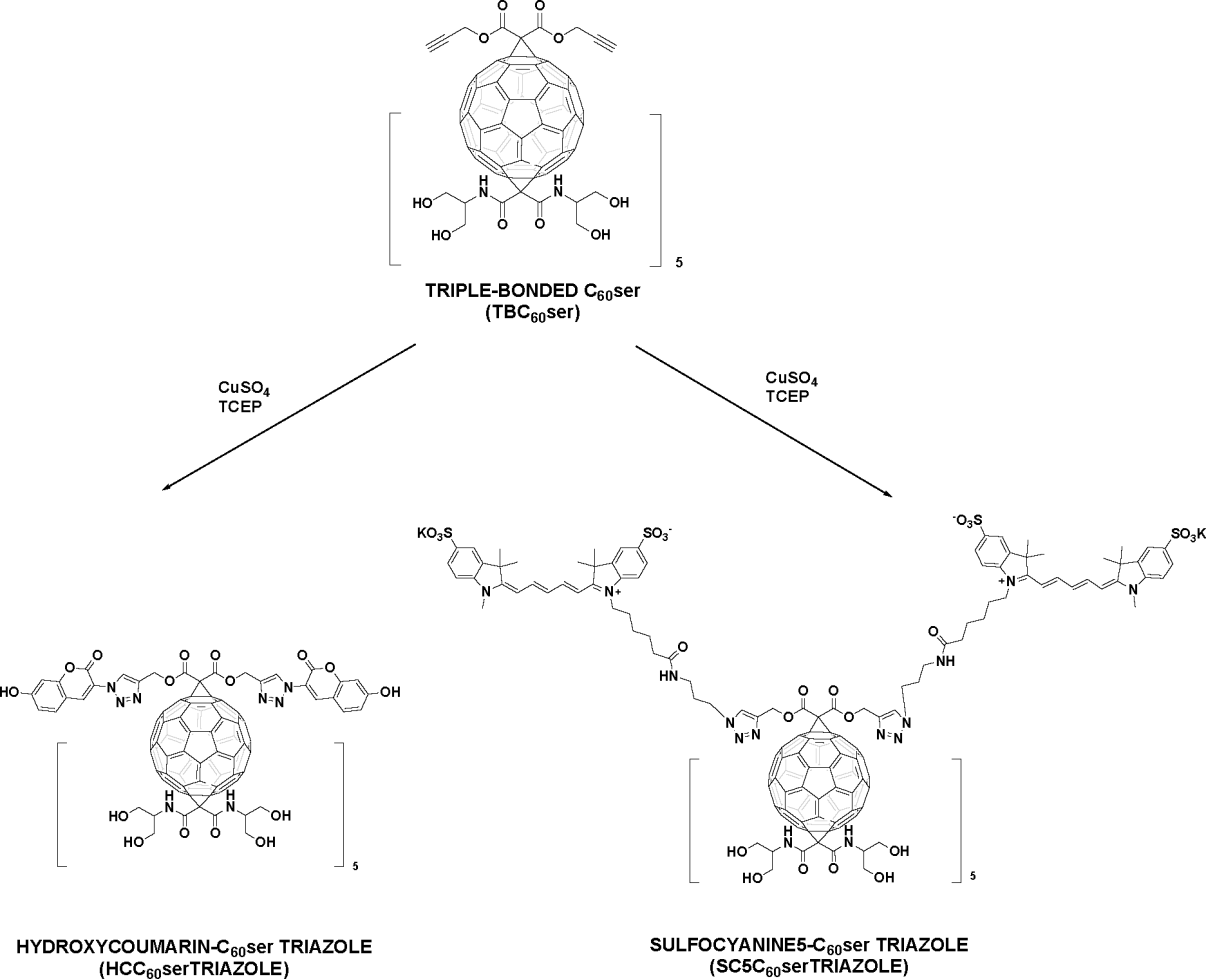
1,3-Dipolar Cycloaddition Reactions between TBC_60_ser and Selected Probes Creating Fluorescent Fullerene Triazoles

Here, we developed a facile method for *in situ* visualization of a water-soluble fullerene nanomaterial, TBC_60_ser, in breast cancer cells MCF 7 (Figure 1). We used the C_60_ser scaffold as a nontoxic and fully water-soluble buckyball, which was previously reported to penetrate through cellular membranes in cancer cells.^19,34^ Therefore, to investigate TBC_60_ser cellular localization, we used two different approaches. First, 7-hydroxy-coumarin azide (HCA) was used as a nonfluorescent precursor that was activated fluorescently only after the formation of [60]fullerene triazole. Second, the bright and photostable sulfo-cyanine 5 (SC5) azide acted as a double control dye, attached to the fullerene scaffold via copper-catalyzed cycloaddition in water (Scheme 1) to doubly confirm the cellular localization of the fullerene nanomaterial. In fact, it was demonstrated here that appropriate *in situ* cellular tagging of [60]fullerene with a triple-bond tag allowed us to visualize engineered fullerene nanostructures in lysosomes of breast cancer cells. Interestingly, during our survey for cellular visualization of nonfluorescent buckyballs, we also synthesized an azide analog of TBC_60_ser, which could be used for biorthogonal approaches with cyclooctyne-derived dyes convenient for copper-free, strain-promoted click reactions. However, our cellular experiments demonstrated that, regardless of the desirable solubility, C_60_ser azide did not pass through the cell membranes and remained in the culture medium (data not shown); thus it did not meet the essential experimental criterion and cannot be used in further investigations. However, it is reasonable to underline the unpredictable issues of altering the pharmacokinetic and physicochemical properties of the nanomaterial during transformation to molecular probes.

## Materials and Methods

### Materials

All of the chemicals used were of reagent-grade quality or better, and the solvents were dried according to literature procedures. The following reagents were used as received: C_60_ (99.5+%, SES Research, USA), propargyl alcohol (Acros Organics), *p*-toluenesulfonic acid monohydrate (Sigma-Aldrich), 1,8-diaza-bicyclo[5.4.0]undec-7-ene (DBU, Sigma-Aldrich), malonic acid (Sigma-Aldrich), CBr_4_ (Sigma-Aldrich), 2-amino-1,3-propanediol (AK Scientific), acetic anhydride (Fisher), *N*-acetylglycine (Acros Organics), 2,4-dihydroxybenzaldehyde (Acros Organics), anhydrous sodium acetate (Sigma-Aldrich), sodium nitrite (Avantor), sodium azide (Sigma-Aldrich), copper sulfate pentahydrate (Avantor), tris(2-carboxyethyl)phosphine (Sigma-Aldrich), and sulfo-cyanine5 azide (Lumiprobe).

### Methods

Nuclear magnetic resonance (NMR) spectra were obtained using a Bruker Advance III 500 MHz NMR spectrometer with tetramethylsilane as the internal standard. Mass spectroscopy (MS) spectra were collected using an electrospray single quad Agilent InfinityLab LC/MSD XT mass spectrometer in the range of 100–3000 Da, equipped with an Agilent HPLC 1260 Infinity II system and SBC18 column (1.8 μm, 2.1 × 50 mm); additional electrospray ionization (ESI) MS measurements were carried out using a Varian 320-MS ESI mass spectrometer. Both ESI-MS measurements were conducted in an acetonitrile/H_2_O/TFA mixture (70/29.9/0.1, v/v). A water-insoluble fullerene monoadduct (**2**) mass measurement was conducted using a Bruker Autoflex II MALDI-TOF mass spectrometer. Attenuated total reflectance Fourier transform infrared (ATR-FT-IR) measurements were taken using a JASCO FT/IR-4600 spectrophotometer equipped with a JASCO ATR PRO ONE kit. Fullerene powders were measured using an ATR ZnSe accessory in the 700–4000 cm^–1^ range. The spectra were recorded using 64 accumulations and at a spectral resolution of 1 cm^–1^. Ultraviolet–visible (UV–vis) and fluorescence spectra were measured on JACSO spectrometers (V-700 and FP 8500 models). Dynamic light scattering and ζ potentials of the fullerene nanomaterial TBC_60_ser were measured using a Zetasizer Nano Instrument (Malvern Panalytical Ltd., UK). High-resolution transmission electron microscopy (HRTEM) observations were performed using a JEOL JEM 3010 microscope operating at a 300 kV accelerating voltage, which was equipped with a Gatan 2k × 2k Orius 833SC200D CCD camera. Chemical analyses of the surface of the fullerenes were performed via X-ray photoelectron spectroscopy (XPS) using a PHI 5700/660 Physical Electronics photoelectron spectrometer with monochromatic Al Kα X-ray radiation (1486.6 eV). The energy of the electrons was measured with a hemispherical analyzer at a resolution of approximately 0.3 eV. Measurements of the photoelectron emission were taken from a surface area with a diameter of 800 μm and at a takeoff angle of 45°. Quantification of the XPS spectra, utilizing peak area and the peak height sensitivity factor, was used for Multipak Physical Electronics analysis. The XPS core-level spectra were fitted using the Doniach–Sunjic method. The final dialysis purification of the water-soluble fullerene nanomaterials was performed on Pall Microsep centrifugal membranes with molecular cut-offs at 1 and 3 kDa (Pall Corporation).

### Synthesis

#### Synthesis of Dipropargyl Malonate

Malonic acid (20 mmol; 2000 mg), *para*-toluenesulfonic acid (*p*-TSA; 0.3 mmol; 60 mg), and 50 mL of toluene were added to a round-bottom flask equipped with a magnetic stirrer and a reflux condenser. Next, a solution of propargyl alcohol (95 mmol; 5380 mg) in 3 mL of toluene was added to the reaction mixture, followed by heating for 48 h at 120 °C. After that time, a brown solution was obtained, which was further extracted with a saturated solution of sodium bicarbonate; organic phases were combined dried over magnesium sulfate, then evaporated on a rotary evaporator to obtain a lightly yellowish, oily dipropargyl malonate. The final product was characterized by NMR spectroscopy (see Supporting Information and Figures S2 and S3).

#### Synthesis of 3-Azido-7-hydroxycoumarin

The nonfluorescent coumarin derivative was synthesized using a modified procedure.^35^ In brief, 2,4-dihydroxy benzaldehyde (20 mmol; 2.76 g), *N*-acetylglycine (20 mmol; 2.34 g), and anhydrous sodium acetate (60 mmol; 4.92 g) were dissolved in 100 mL of acetic anhydride and heated under reflux for 4 h. After this time, the reaction mixture was poured into an ice container, and the resulting yellow solid of peracetylated 3-amino-7 hydroxycoumarin was filtered under reduced pressure. The intermediate was used for further reactions without additional purification. In order to hydrolyze the acetyl protecting groups from 3-amino-7 hydroxycoumarin and introduce the azide group in the 3-position of the coumarin, a hydrolysis reaction followed by the formation of diazonium salt was performed. For this purpose, the previously obtained intermediate was heated under reflux in a solution of concentrated HCl and ethanol at a 2:1 volume ratio (20 mL of 35% HCl and 10 mL of 95% C_2_H_5_OH) for 1 h. The reaction mixture was allowed to cool, and 20 mL of cold water was added to dilute the solution. The reaction mixture was then cooled in an ice bath, and sodium nitrite (40 mmol; 2.760 g) was gradually added before stirring for another 5–10 min. Then, sodium azide (60 mmol; 3.900 g) was added in small portions. After stirring for 15 min at room temperature, the resulting precipitate was filtered, washed with water, and then dried *in vacuo* to give the final azide as a brown solid, with a melting point of 121 °C (lit: 118–120 °C).^35^ The final compound was characterized by NMR and UV–vis spectroscopies, and its lack of fluorescence properties was confirmed in cellular experiments.

#### [60]Fullerene Monoadduct (**2**)

The [60]fullerene (0.5 mmol; 360 mg) was dissolved in 400 mL of dry, degassed toluene using an ultrasonic bath (20 min). To obtain a purple solution of C_60_, dipropargyl malonate (0.44 mmol; 80 mg) and CBr_4_ (0.63 mmol; 210 mg) were added with intense stirring. Next, a DBU solution (0.625 mmol; 95 mg) in 7 mL of toluene was added dropwise to the reaction mixture. The reaction mixture was stirred for 3 h at room temperature and monitored by the thin-layer chromatography (TLC) technique. Upon completion of the reaction, a brown solution of [60]fullerene monoadduct was obtained, which was first purified by pouring the reaction mixture through a silica plug to remove mostly unreacted [60]fullerene, and then a brownish monoadduct fraction was further purified on a column using a toluene/dichloromethane 1/1 (v/v) eluent, followed by evaporation on a rotary evaporator. A lightly brownish solid was obtained (102 mg, 22% yield), which was further characterized by NMR and FT-IR spectroscopies and MALDI-TOF spectrometry. The [60]fullerene monoadduct (**2**) spectral characterization was in accordance with literature describing triple-bonded [60]fullerene derivatives (with 3-butynyl fragments).^36^ The spectral characterization and MALDI-TOF mass spectrometry of compound (**2**) can be found in the Supporting Information (Figures S8 and S9).

#### The [60]Fullerene Hexakisadduct (**3**) and Its Water-Soluble Analog (**4**)

A large-scale synthesis of peracetylated diserinol malonate was published by our group previously.^37^ The [60]fullerene monoadduct (**2**) (0.2 mmol; 144 mg) was dissolved in a mixture of 10 mL of dry methylene chloride and 100 mL of dry toluene while stirring vigorously at room temperature in a nitrogen atmosphere. The peracetylated diserinol malonate was added to a fullerene solution (2 mmol; 836 mg) with an excess of carbon tetrabromide (4 mmol; 1324 mg). Next, a solution of DBU in chloroform was prepared by dissolving 1,8-diazabicyclo[5.4.0]undec-7-ene (2.4 mmol; 362 mg) in 3 mL of chloroform, which was added in 0.5 mL portions every 60 min, and the reaction mixture was stirred at room temperature for 72 h, observing a color change of the solution to brown-reddish. The final fullerene hexakisadduct (**3**) was purified using gradient flash column chromatography with dichloromethane and methanol as eluents (starting from 99:1 and finishing with 50:50 v/v), resulting in the formation of a brownish, oily liquid in 27% yield. The water-insoluble fullerene nanomaterial (**3**) was subsequently deprotected from acetyl protecting groups using the HCl-1,4-dioxane methodology developed earlier.^37^ In brief, the peracetylated [60]fullerene derivative (**3**) was dissolved in 18 mL of 1,4-dioxane, and 3 mL of concentrated HCl was added to the brownish solution of fullerene nanomaterial and stirred for 7 days at room temperature. After that time, the final product was purified by three cycles of dialysis of an aqueous solution of (**4**) using a centrifugal membrane (molecular weight exclusion limit = 1.0 kDa; Nanosept, Pall Corporation, USA), which was then lyophilized and stored at −20 °C.

#### *In Vitro* Cu-Catalyzed Click Reactions using TBC_60_ser and Organic Azides (HCA and SC5)

Before the cellular experiments, all novel fullerene triazoles were synthesized using copper(I)-catalyzed click reactions. In general, 5 mg of TBC_60_ser was dissolved in 10 mL of DI water (and 5 mL of DMSO in the case of HCA reaction), and 1 mg of the appropriate organic azide was added to the fullerene solution with the addition of 0.1 mmol CuSO_4_·5H_2_O and 0.1 mmol tris(2-carboxyethyl)phosphine (TCEP) as a reducing agent; the reaction mixture was further stirred at room temperature for 6 h. After that time, fullerene triazoles were purified using centrifugal membranes with 1-kDa cut-offs (Pall Corporation, USA) and characterized using ESI-MS, UV–vis, and FT-IR spectroscopy.

### Biological Studies

#### Cell Culture and Cytotoxicity

The human breast carcinoma cell line (MCF-7) was purchased from ATCC. The normal human dermal fibroblasts cell line (NHDF) was obtained from PromoCell. MCF-7 was cultured in Dulbecco’s modified Eagle’s medium (DMEM) supplemented with 10% heat-inactivated fetal bovine serum (FBS, all from Sigma-Aldrich) containing a 1% v/v mixture of antibiotics (i.e., penicillin/streptomycin, Gibco). The DMEM for the NHDF were supplemented with 15% noninactivated FBS and antibiotics. The cells were grown under standard conditions at 37 °C with a 5% CO_2_ humidified atmosphere.

The MCF-7 and NHDF cells were seeded into 96-well plates (Nunc) at a density of 5000 cells per well and incubated at 37 °C for 24 h for cytotoxicity experiments. The next day, the complete DMEM was replaced with solutions of tested fullerene nanomaterials, azides, or copper(II) sulfate pentahydrate at various concentrations. A cytotoxicity assay was performed after 72 h of incubation using CellTiter 96AQueous One Solution-MTS (Promega) according to the supplier’s protocol. In short, the solutions of tested compounds were removed, and 100 μL of DMEM (without FBS or phenol red) with 20 μL of the MTS reagent were added to each well and incubated at 37 °C for 1 h. Then, the samples’ absorbance was measured at 490 nm using a multiplate reader (Synergy 4, BioTek). The results were calculated as the percentage of the control (untreated cells) and estimated as the inhibitory concentration (IC_50_) values (using GraphPad Prism 9). Each experiment was performed three times.

#### Cellular Staining

Before cellular staining experiments, MCF-7 cells were seeded onto coverslips at a density of 120,000 cells per slide and incubated at 37 °C for 48 h. Then, the DMEM was removed, and solutions of the fullerene nanomaterial (TBC_60_ser, 468 μM = 1 mg/mL), SC5 (25 μM), and HCA (25 μM) were added and further incubated for 2 h. Additionally, nuclei were stained with Hoechst 33342 (Invitrogen). Then, the cells were washed three times with PBS and mounted with DMEM without FBS or phenol red. The cellular staining results were immediately observed after excitation at 386 nm/438 and 650 nm (Cy5 filter) using the CellInsight CX7 High Content Analysis Platform (ThermoFisher).

#### *In Situ* Click Reactions and Cellular Colocalization Studies

MCF-7 cells were seeded in the same manner as described in Cellular Staining section. Then, the DMEM was removed, and the solution of fullerene TBC_60_ser (468 μM) was added and further incubated overnight. After this time, the cells were washed twice with PBS and incubated with click reaction reagents SC5 (25 μM) or HCA (25 μM), CuSO_4_ (1 mM), and TCEP (1 mM) for 2 h at 37 °C. Additionally, nuclei were stained with Hoechst 33342 (Invitrogen), mitochondria with MitoTracker Green or Orange, and lysosomes with LysoTracker Yellow HCK-123 according to previously described protocols.^38^ The MCF-7 cells were washed three times with PBS and mounted with DMEM without FBS or phenol red. Cellular imaging was performed using the CellInsight CX7 High Content Analysis Platform under an appropriate filter for the click reaction or dyes used and a 40× objective. The fluorescence images were processed using ImageJ software 1.41 (Wayne Rasband, National Institutes of Health, Bethesda, MD, USA). The Manders’ and Pearson’s coefficients, which were used to show the colocalization triazole derivatives of TBC_60_ser with specific-organelle trackers, were calculated using the ImageJ plugin “JACoP.”

## Results and Discussion

The synthetic approach to [60]fullerene derivatives is mainly based on two synthetic approaches, which rely on classical Bingel–Hirsch cyclopropanations and Prato cycloadditions. The aforementioned methodology provides an opportunity to create fully water-soluble fullerene nanotherapeutics that are decorated with solubilizing addends.^39^ In the case of the Bingel–Hirsch reaction, only two regioisomers are in the purview of the medicinal chemist: [60]fullerene monoadducts and corresponding hexakisadducts, which can exist only as one regioisomer and can be easily recognized by ^13^C NMR measurements.^40^ A plethora of isomers of fullerene derivatives could be formed in the case of other regioisomers (bis-, tris-, tetrakis-, and pentakis-adducts), observed in their purified form only when complicated and laborious separation techniques are applied. An example of such strenuous procedures is the work of Shi and co-workers, who separated 19 structural isomers of bisPCBM [60]fullerene.^41^

**Scheme 2:**
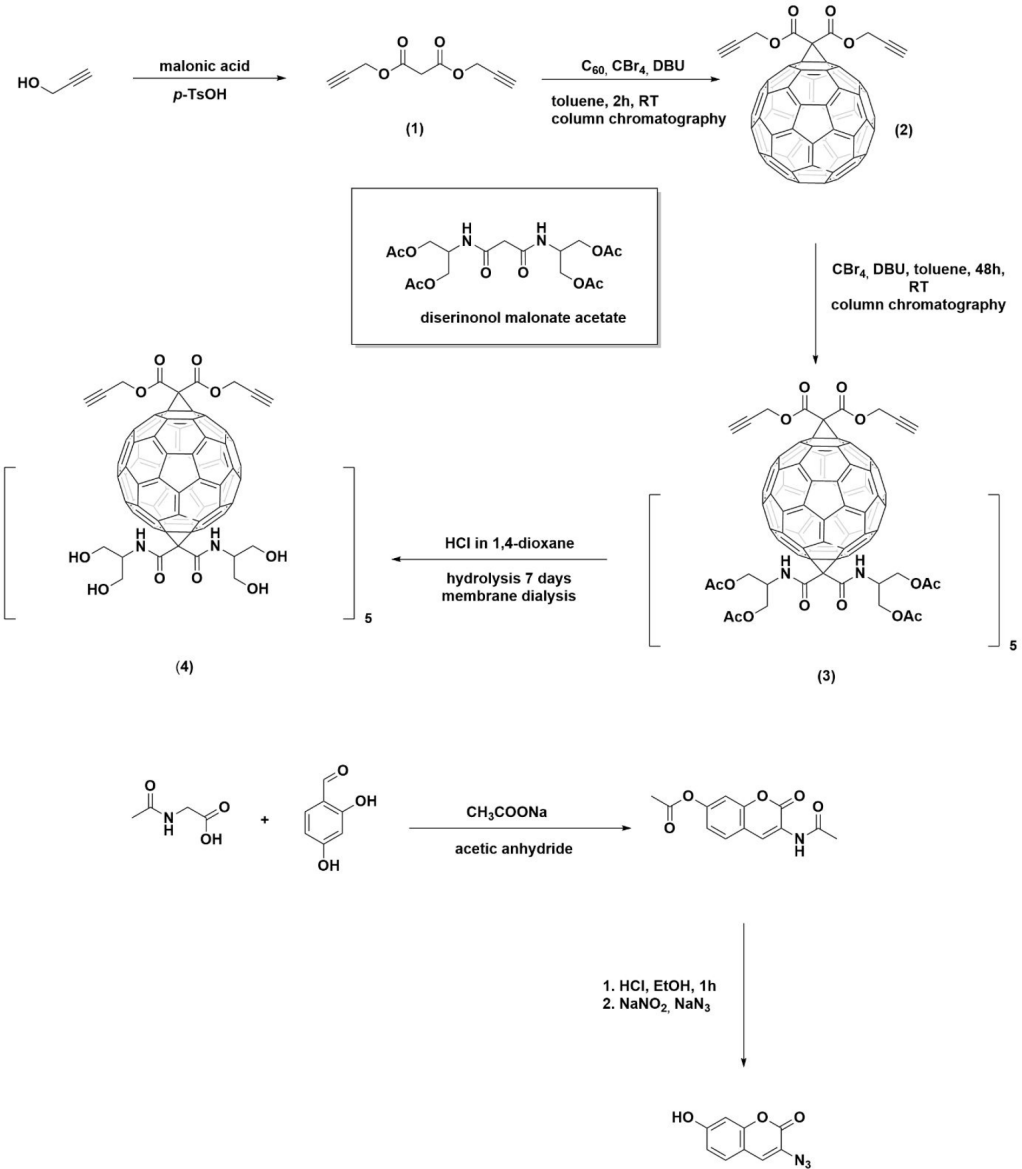
Synthetic Strategy for Obtaining Water-Soluble Fullerene Nanomaterial TBC_60_ser and Nonfluorescent Dye 3-Azido-7-hydroxycoumarin

Here, we developed a robust methodology to create a fully water-soluble [60]fullerene hexakisadduct containing two different malonate addends: one containing the triple bonds and one with diserinol malonate units as a solubilizing scaffold (see the synthetic protocol depicted in Scheme 2). The dipropargyl malonate was synthesized using a simple esterification procedure, and its spectroscopic characteristics are presented in the Supporting Information (Figures S2–S5). The fullerene monoadduct (**2**) was obtained by mixing the buckyball (C_60_) with dipropargyl malonate in the presence of CBr_4_ and DBU, using time-controlled (3 h) Bingel–Hirsch cyclopropanation to avoid the formation of bis- and trisadducts. The ^1^H NMR spectra showed characteristic signals of methine protons close to 2.49 ppm, whereas symmetry was confirmed by ^13^C NMR, with 15 signals of fullerene sp^2^ carbons and one sp^3^ carbon appearing close to 70 ppm (Supporting Information, Figure S8). The structure of the created fullerene derivative (**2**) was additionally confirmed by MALDI-MS, which confirmed that the mass of the fullerene monoadduct was 897 Da (Figure S9), where the observed molecular ion peak had an *m*/*z* value matching that of the calculated monoisotopic mass. Further functionalization of the triple-bonded monoadduct to the water-soluble *T*_*h*_ symmetrical hexakis-adduct was carried out in a second cyclopropanation reaction with peracetylated diserinol malonate as a water-solubilizing scaffold. By monitoring the progress of the reaction (72 h) using TLC and the slow addition of DBU over 6 h, we were able to obtain the peracetylated [60]fullerene hexakisadduct (**3**). This was purified using column chromatography and immediately hydrolyzed to obtain the highly water-soluble fullerene nanomaterial (**4**), which was further characterized using NMR, IR, and XPS spectroscopies, and its mass was confirmed by ESI-MS. The ^13^C NMR spectrum of *T*_*h*-_symmetrical [60]fullerene derivative (**4**) is shown in Figure S1, and signal contributions from two C_60_-sp^2^ carbons (144 and 141 ppm) and one C_60_-sp^3^ carbon (69 ppm) were clearly observed. Two different signals from the carbonyl groups that are present in the fullerene nanomaterial (**4**) were also noticeable between 165 and 170 ppm as well as two characteristic signals of triple-bonded carbons at 78 and 77 ppm. As depicted in Figure S10, a molecular ion peak at 2141 Da was observed for a water-soluble hexakis-fullerene derivative (**4**), which corresponds to a [M + 2H]^+^ cation that could be formed in eluent containing 0.1% TFA (calculated mass for fullerene [**4**]: 2139 Da) in fullerene decorated with hydroxyl groups; thus, the spectroscopic data, in combination with mass spectrometry, clearly confirmed the creation of the symmetrical [60]fullerene derivative (**4**).

**Figure 2:**
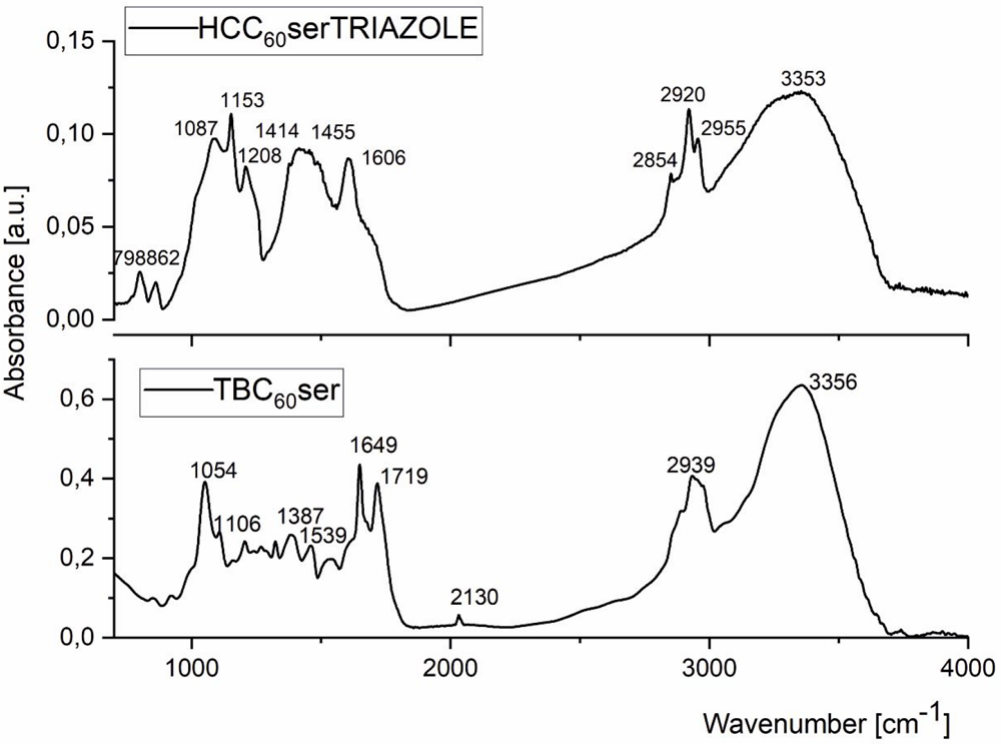
FT-IR spectrum of TBC_60_ser and its fluorescent triazole derivative HCC_60_serTRIAZOLE.

FT-IR spectroscopy is a convenient method to confirm the presence of functional groups that are attached to engineered carbon nanomaterials. When studying the formation of fullerene triazoles, FT-IR could also be helpful to confirm that no unreacted terminal alkyne residues (signals around 2100 cm^–1^) remain in the final products. In the case of fullerene nanomaterial (**4**), two different types of carbonyl groups are present in the molecule due to two different types of malonate addends connected to the buckyball scaffold: dipropargyl malonate (ester) and diserinol malonate (secondary amide). Here, the characteristic absorbance of two different carbonyl groups present in fullerene hexakisadduct (**4**) was observed at 1649 and 1719 cm^–1^ (Figure 2), which correspond to stretching vibrations of carbonyl moieties *v*(C=O). The strong intensity band at 1649 cm^–1^ is linked to stretching vibrations of the carbonyl group present in the secondary amide, whereas a band near 1539 cm^–1^ is characteristic of the in-plane N–H bends of the secondary amide group.^42^ On the other hand, an intense band near 1719 cm^–1^ is caused by unsaturated ester fragments in the structure of TBC_60_ser, namely, dipropargyl malonate units (see Figure S5). Diagnostic signals from the terminal bond present in TBC_60_ser are easily found as weak bands close to 2130 cm^–1^ (C≡C); however, C–H stretch signals (3330–3270 cm^–1^) are not easily observed in functionalized fullerene derivatives with many OH groups and in the presence of hydrogen bonds. Intense and vast bands near 3300 cm^–1^ confirm the presence of OH stretching vibrations with additional bending modes (δC–OH and δOH near 1380 cm^–1^).

Before performing the cellular 1,3-dipolar cycloadditions, we confirmed the formation of two different fullerene triazoles (HCC_60_serTRIAZOLE and SC5C_60_serTRIAZOLE, Scheme 1) using spectroscopic techniques (i.e., FT-IR and UV–vis) as well as ESI-MS. The FT-IR spectrum of HCC_60_serTRIAZOLE is presented in Figure 2 and compared to the parent structure-TBC_60_ser. The analysis of this spectrum provides evidence for the changes in the structure of starting fullerene nanomaterial, TBC_60_ser, with an apparent absence of characteristic signals from the triple-bond function (near 2130 cm^–1^), indicating a successful 1,3-dipolar cycloaddition and the formation of triazole. The broad- and medium-range signal at 1606 cm^–1^ could also be identified as a N=N stretching mode from the triazole ring in combination with stretching vibrations of secondary amide *v*(C=O); however, additional weak IR stretches from the C=CH groups of the triazole ring are not visible due to strong and broad OH signals in the 3000–3500 cm^–1^ range. Additional confirmation of the formation of coumarin-based triazole came from MS analysis. As depicted in Figure S11, one can observe a characteristic molecular ion peak at 2542 Da, which could be associated with our [60]fullerene derivative HCC_60_serTRIAZOLE (calculated theoretical mass = 2543 Da). Additional ESI-MS experiments performed using a higher voltage (300 mV) revealed a fragmentation ion at 2325 Da [M + Na]^+,^ which could be associated with a fragment with only one triazole attached to the fullerene scaffold (calculated mass for M + H]^+^ ion = 2302 Da, Figure S12). The more challenging structure of fullerene nanomaterial SC5C_60_serTRIAZOLE was also successfully confirmed using spectroscopic methods (i.e., UV–vis and FT-IR), supplemented by EIS. As in the case of terminal alkynes, the infrared spectrum of organic azides has a diagnostic range close to 2100 cm^–1^, which is presented in the case of the second substrate for click reaction: sulfo-cyanine5 azide dye (Figure S6, band near 2097 cm^–1^). In the FT-IR spectrum of SC5C_60_serTRIAZOLE (Figure S7), the strongest and broad stretches located at 1615 cm^–1^ corresponded to the plethora of secondary amide groups presented within the engineered structures of the fullerene nanomaterial, with an absence of azide groups. The presence of the sulfo group in the structure of fullerene triazole could be also correlated with signals at 1040 and 1100 cm^–1^ (S=O), whereas the hydroxyl groups from several diserinol fragments are shown as an extensive band near 3300 cm^–1^, making it impossible to observe triazole stretches. The molecular peak of our fullerene nanomaterial, SC5C_60_serTRIAZOLE, was not detected at 3663 Da, [M + H]^+^ due to the limits of our ESI detector (3000 Da); however, additional fragmentation analyses confirmed the successful 1,3-dipolar cycloaddition and formation of triazole. As depicted in Figure S13 for a higher applied voltage (300 V), one could observe a fragmentation ion at 2826 Da, corresponding to the formation of a specific one-armed sulfo-cyanine5 cation with cleaved fragment sulfo-cyanine5 connected via an ester bond. Interestingly, the mass observed at 2137 Da was derived from the parent structure by cleaving two sulfo-cyanine5 units and the formation of a malonic acid ethyl ester derivative (Figure S12).

**Figure 3:**
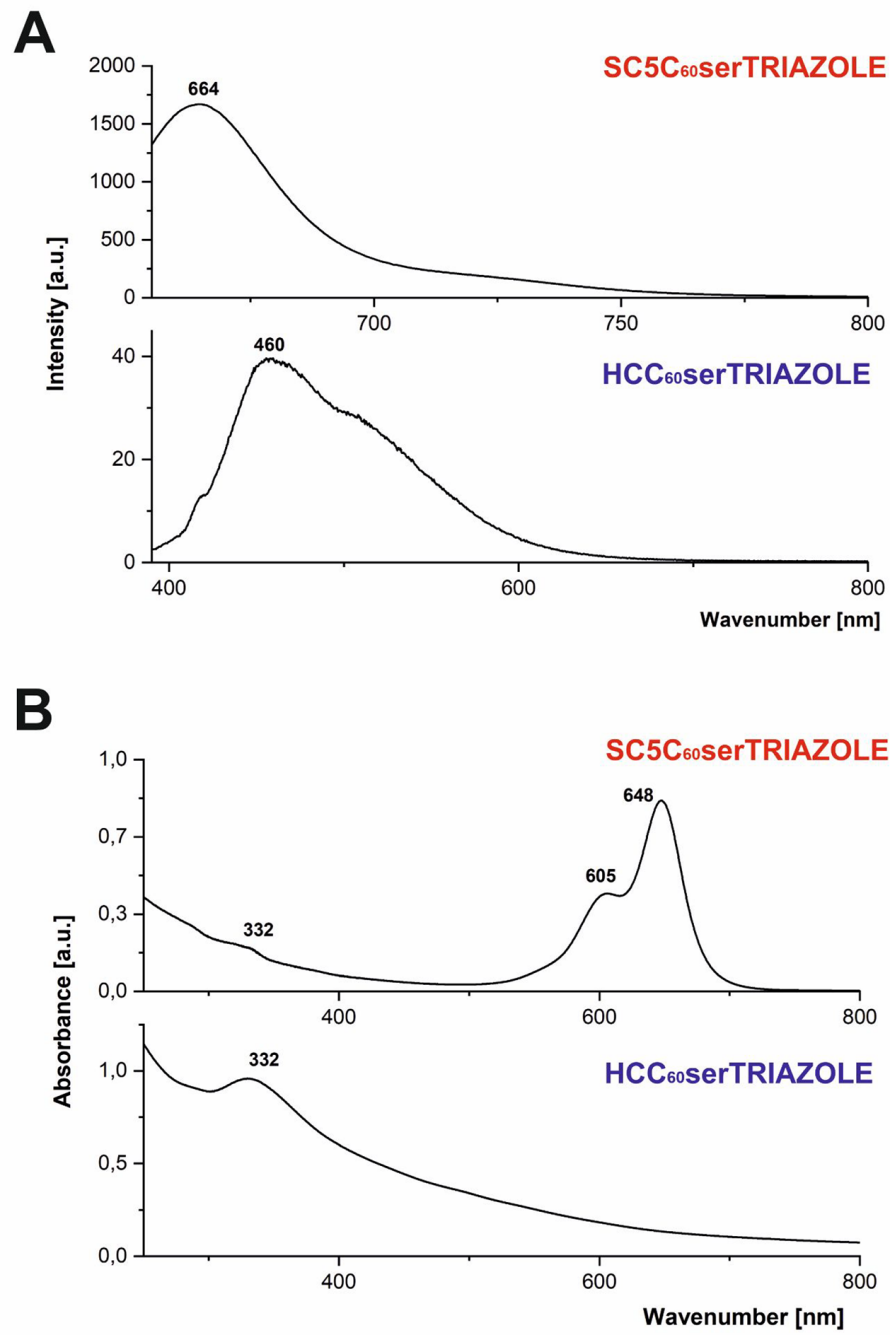
Fluorescence (A) and UV–vis (B) spectra of fullerene-based fluorescent triazoles HCC_60_serTRIAZOLE and SC5C_60_serTRIAZOLE (DI water, *c* = 0.01 mg/mL).

According to our theoretical assumptions, after the *in situ* click reactions, the formed fullerene triazoles should be fluorescent and easily visualized in the cellular environment. The UV–vis and fluorescent spectra of organic azides SC5 and HCA are presented in Supporting Information (Figures S16–S19). As shown in Figure 3A, the electronic spectra of fullerene triazoles are presented with a characteristic maximum from a fullerene fragment at 332 nm and strong sulfo-cyanine fragments observed at 605 and 648 nm. The emission spectra of the desired fullerene-based triazoles in water are depicted in Figure 3B, confirming the formation of blue-emitting HCC_60_serTRIAZOLE (460 nm) and red-emitting SC5C_60_serTRIAZOLE (664 nm). In this context, a crucial question should be asked—whether the presence of albumin proteins and protein corona formation on the surface of fullerene nanomaterials in the cellular milieu would quench their fluorescence. Further cellular *in situ* click reactions of water-soluble TBC_60_ser were carried out to address this relevant question.

**Figure 4:**
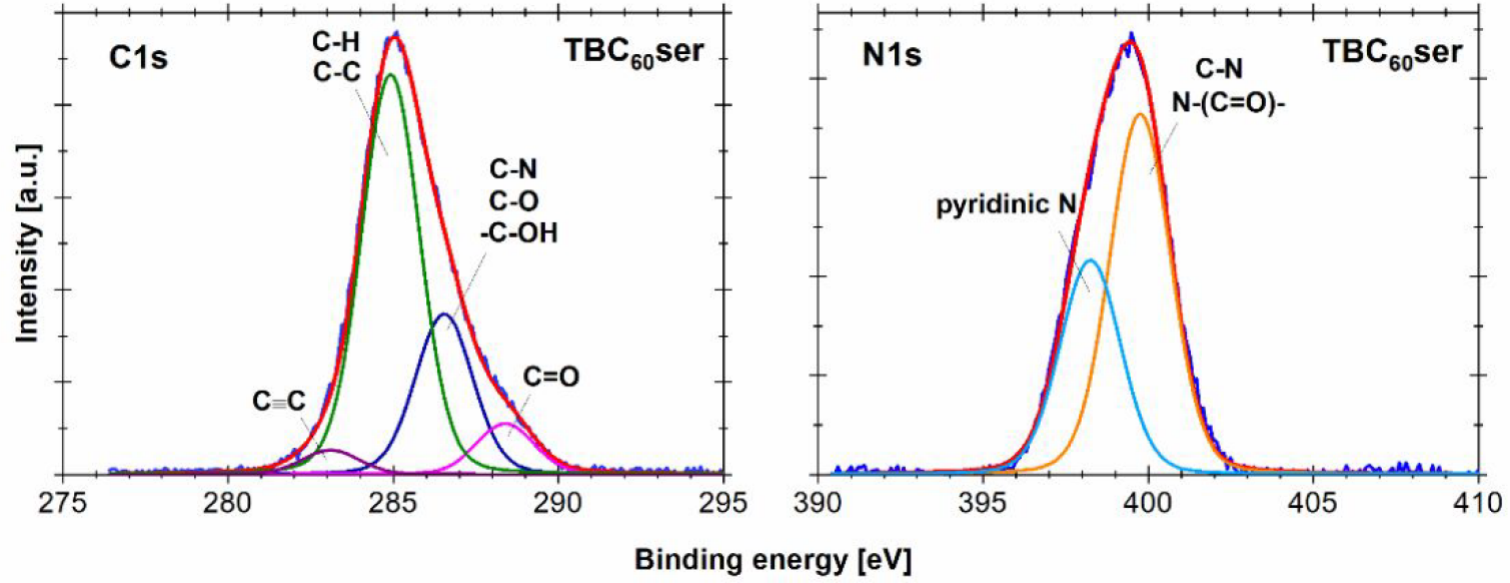
XPS profiles (C 1s and N 1s) of fullerene nanomaterial TBC_60_ser.

The photoelectron spectroscopy technique (XPS) was used to examine the electronic structure and composition of TBC_60_ser. Atomic concentration calculations were made based on the ratio of each of the compounds to the sum of all the compositional elements. The photoemission lines of C 1s, O 1s, and N 1s were deconvoluted after background subtraction to determine possible chemical bonds in the examined sample (Figures 4 and S15). Table S1 shows the chemical composition, atomic concentration, and percentage contributions of chemical state for a particular element. The C 1s peak can be deconvoluted into four lines, corresponding to carbon atoms existing in different functional groups. The most intensive line at 248.9 eV was characteristic for graphitic carbon (i.e., C–C or C–H). The second line at 286.5 eV was related to oxygen- and nitrogen-containing groups (i.e., C–O, C–N, or −C–OH), while carbonyl groups C=O were represented by the third line at 288.3 eV.^43^ The O 1s spectrum revealed three compositional lines that were assigned to carbonyl groups (C=O) at 531.9 eV, carboxyl groups (O–C=O) at 533.2 eV, and quinones at 530.5 eV.^44^ The N 1s spectrum consisted of two components: The peak located at 399.8 eV was ascribed to C–N and N–(C=O)– bonds, while the peak located at 398.3 eV was ascribed to basic nitrogen (pyridinic type).^45,46^

Further studies were conducted to determine the occurrence of carbon–carbon triple bonds within the TBC_60_ser fullerene nanomaterial. The reported literature data indicated the presence of a C 1s line at a binding energy of 283 ± 0.2 eV, where carbyne bonds (triple bond between carbon and the transition metal) were detected.^47^ Additionally, the C≡C alkynic bond for methylacetylide (CH_3_–C≡C–Ag) was revealed at binding energies of 283.3 and 283.6 eV in a study of the electronic structure of unsaturated C_3_H_3_ groups adsorbed on a silver surface.^48^ For acetylide species (H–C≡C−), the binding energy assigned to the C≡C bond was measured at 283.1 eV.^49^ Because the carbon–carbon triple bond occurs in the structure of TBC_60_ser fullerene in small amounts, the intensity of the photoemission line assigned to this state should be relatively low. Moreover, based on the above-mentioned literature data, this line should be located at a relatively low binding energy, and its detection may be difficult due to its proximity to the most intense C 1s line of the compound (C–C/C–H). We observed a chemical state with a binding energy at 283.1 eV, which should be related to the presence of a carbon–carbon triple bond. The atomic concentration calculations and deconvoluted C 1s line indicated that 2.8% of carbon atoms formed triple bonds. These results correlated well with the number of bonds present in the examined structure, where the estimated concentration of carbon in a triple bond should be approximately 3.7 at%. The slightly lower value of detected triple-bonded carbons might be the result of the presence of surface contamination.

**Figure 5:**
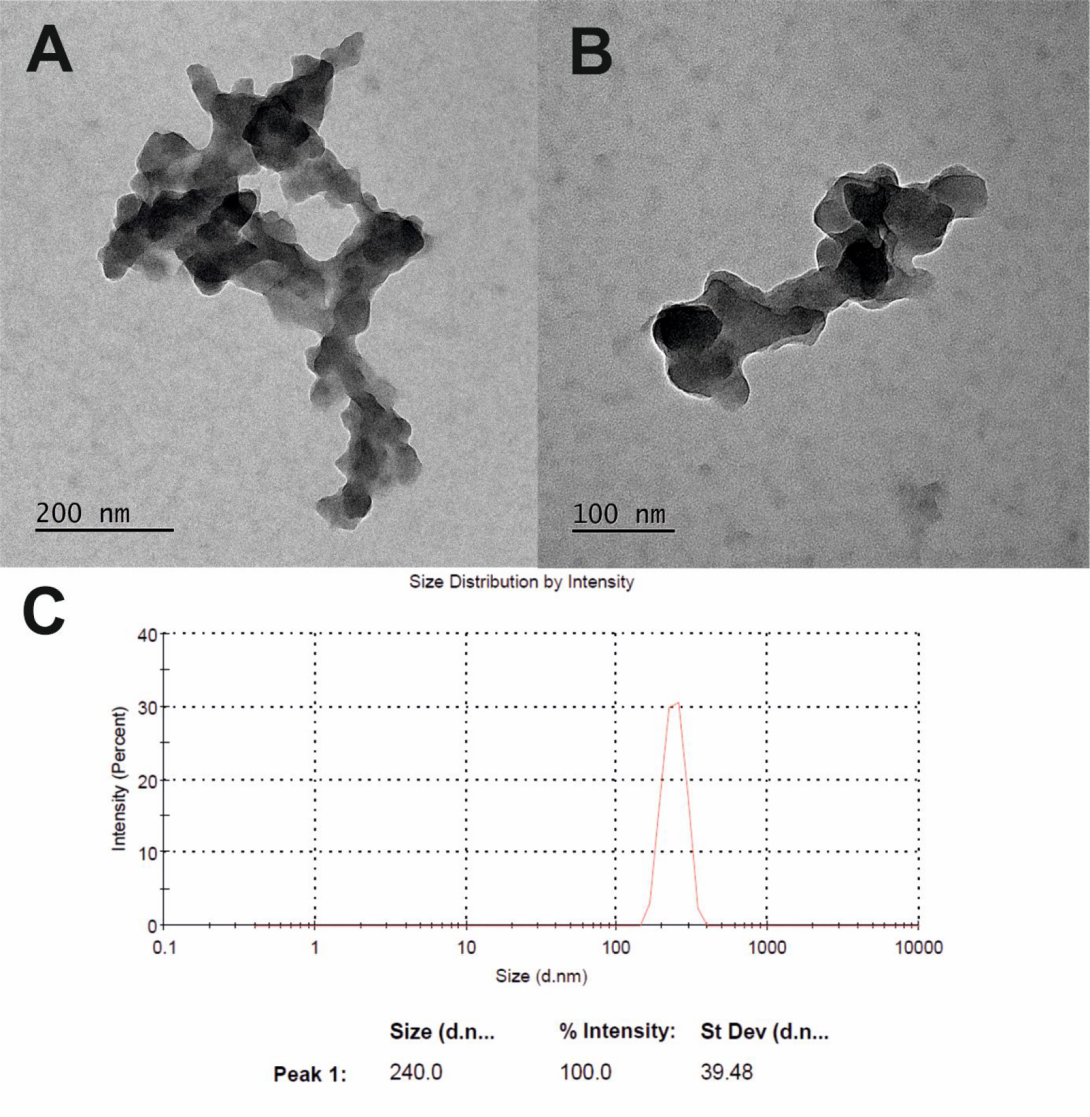
(A, B) Images of fullerene nanomaterial TBC_60_ser visualized using TEM. (C) DLS measurement of TBC_60_ser.

Further characterization of TBC_60_ser was performed using TEM microscopy and dynamic light scattering (DLS) measurements, as depicted in Figure 5. As previously reported by Wilson et al., the malonodiserinolamide [60]fullerene derivative (C_60_ser) formed aggregates in water, which were in dynamic equilibrium with a small percentage of single C_60_ser molecules.^34^ During the analysis of TEM images of TBC_60_ser, it was revealed that it formed fluffy-like aggregates ranging from 100–500 nm, but smaller aggregates were also observed (Figures 5A,B). A similar observation was reported by Wilson when studying C_60_ser but using scanning electron microscopy.^34^ For the DLS of TBC_60_ser, the peaks were concentration dependent; upon increasing the concentration, larger aggregates were also observed (for a concentration above 1 mg/mL, peaks above 1 μm were <1% of the detected fullerene aggregates). Furthermore, the average size of [60]fullerene derivative TBC_60_ser agreed with the hydrodynamic diameter determined by DLS in DI water (240 nm, Figure 5C). To better understand the interactions of the triple-bonded buckyball in the cellular milieu, its ζ potential was measured, showing a stable negative charge (−34.8 mV, Figure S14), which confirmed that it was stable in water solutions (ζ potential higher than ±30 mV). It should also be mentioned that charged nanoparticles have higher cell internalization and faster opsonization rates than electrically neutral particles; however, negatively charged nanoparticles are slowly incorporated by cells.^50^

In the final stages of our research, we performed biological studies to verify the possibility of forming adducts of triazole derivatives of fullerene with dyes hydroxycoumarin azide (called HCC_60_serTRIAZOLE) and sulfo-cyanine azide5 (called SC5C_60_serTRIAZOLE) in the cellular environment using a copper-catalyzed click reaction. The basis of using all these components for cell labeling by click chemistry was their low toxicity. Because of this, we first investigated the cytotoxicity of the investigated fullerene nanomaterial (TBC_60_ser), hydroxycoumarin azide (HCA), sulfo-cyanine azide5 (SC5), and copper sulfate (CuSO_4_) on a human breast cancer cell line (MCF-7). The cells were treated with a wide range of concentrations of compounds that were tested for 72 h. After this time, the cytotoxicity was determined using the colorimetric method (i.e., an MTS assay based on tetrazolium salt). As presented in Table S2 in the Supporting Information, TBC_60_ser at a concentration of 468 μM (1 mg/mL) did not affect cell viability or cell number during the long-term assay. We report similar results for both tested dyes, where the concentration (25 μM) used for the click reaction did not induce a cytotoxic effect. TBC_60_ser was also nontoxic on normal cells.

**Figure 6:**
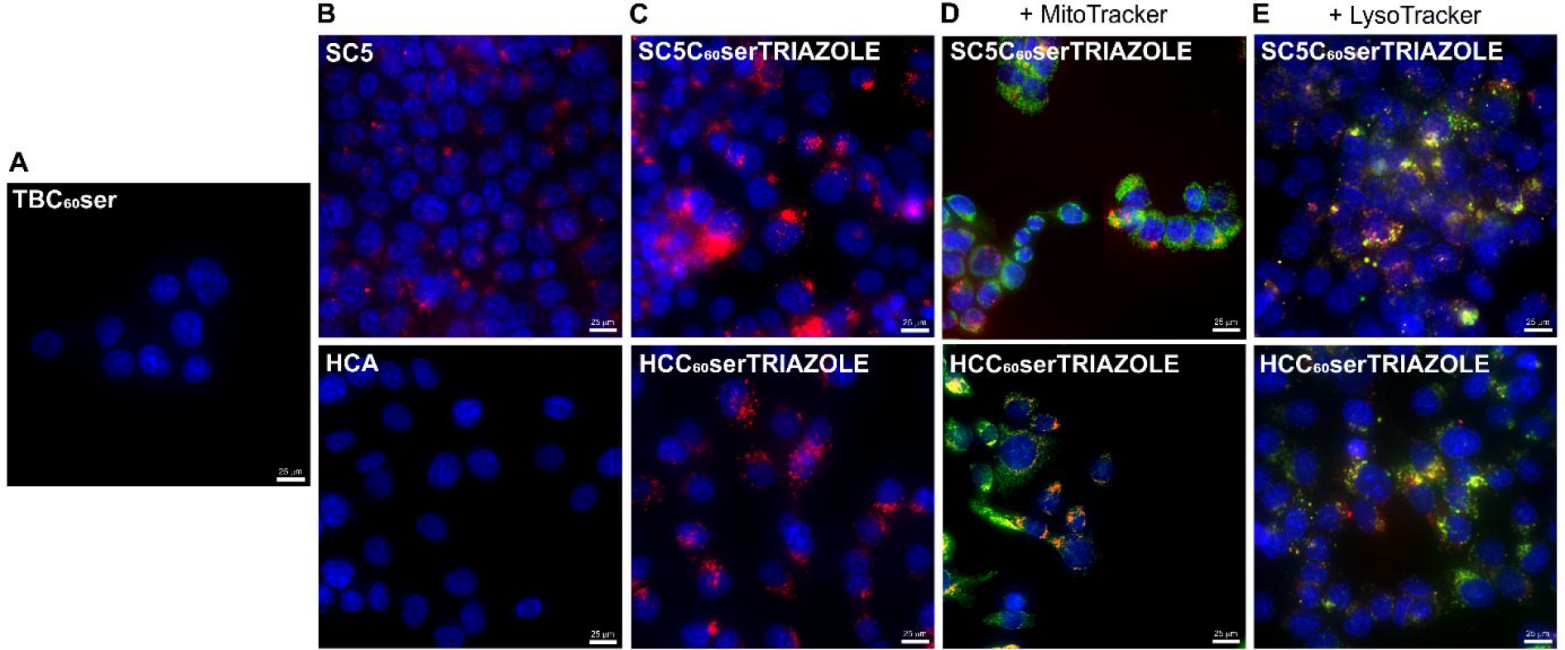
Cellular colocalization study of fullerene nanomaterial TBC_60_ser (A), dyes: SC5 and HCA (B), and its triazole derivatives (C–E) in breast cancer (MCF-7) cell line. Cell nuclei are colored blue, mitochondria/lysosomes in green, and SC5 and fullerene adducts (SC5C_60_serTRIAZOLE and HCC_60_serTRIAZOLE) in red. HCC_60_serTRIAZOLE is labeled red in ImageJ, whereas it is green in the live image. Scale bars = 25 μm.

To evaluate the behavior of TBC_60_ser, HCA, and SC5 ligands in the cellular environment, we performed a series of live-cell imaging experiments. The results are shown in Figure 6A,B. As expected, fullerene and hydroxycoumarin azide did not show any fluorescence after excitation at 386 and 438 nm, respectively. Indeed, according to our assumption, the hydroxycoumarin azide should be activated only after attachment to TBC_60_ser. On the other hand, for sulfo-cyanine azide5, after excitation at 650 nm, we recorded a red fluorescence signal in the area adjacent to the cell nucleus. Next, we optimized our two approaches for a cellular copper(I)-catalyzed click reaction by testing different variants of doses and incubation times of the components used to label the cells (approaches are presented in Scheme 1). Finally, we performed experiments in which we incubated the MCF-7 cells with 468-μM TBC_60_ser for 24 h, followed by another 2 h incubation with 25 μM HCA or SC5 dye. The cell images were acquired immediately after labeling, and the fluorescence signals were registered after excitation at 386 nm/438 or 650 nm (Cy5 filter), depending on the visualization approach used. As shown in Figure 6C, the cells were successfully labeled through the copper-catalyzed reaction between the nonfluorescent fullerene (TBC_60_ser) and SC5 or HCA azides, which resulted in highly fluorescent triazole derivatives: SC5C_60_serTRIAZOLE and HCC_60_serTRIAZOLE. The formed HCC_60_serTRIAZOLE adduct provided a green fluorescent signal in the real live image. However, it is marked with red in Figure 6 for better clarity. The localization of fullerene triazoles in cells was determined by costaining with cell-trackers binding to mitochondria and lysosomes (Figure 6D,E). As a result of this staining, it was observed that both compounds SC5C_60_serTRIAZOLE and HCC_60_serTRIAZOLE had a higher tendency to accumulate in lysosomes (Figure 6E). The images generated from the combined channels of the tested compounds and the LysoTracker clearly show multiple overlapping areas of localization (indicated in yellow). These results appear to be consistent with a previous report on the cellular uptake of fullerene nanomaterials into cells via the clathrin-dependent endocytic pathway and their distribution in lysosomes.^51^

Similarly, studies using high-contrast optoacoustic and THG imaging techniques confirmed the localization of the C_70_@lysozyme complex inside lysosomes of HeLa cells.^22^ In addition, the subcellular localization and tendency of C_60_ fullerenes to accumulate in lysosomes may be explained by their surface charge. Recently, Ma et al.^52^ revealed that anionic C_60_-(EDA-EA) with a ζ potential of −15 mV was preferentially transported into lysosomes. In contrast, cationic C_60_-EDA (+13 mV), under the influence of a negative membrane potential in the cell, was able to enter cells more rapidly and enrich mitochondria.^52^ On the other hand, some reports indicated that C_60_ fullerenes with high electronegativity may have a higher affinity for mitochondria due to a protonated pool in the intermembrane space.^53^ Interestingly, our tested compounds may also bind to mitochondria to a much lesser extent (Figure 6D).

We performed a quantitative evaluation to validate our observations by calculating the Pearson correlation coefficient (PCC) and Mander’s overlap coefficient (MOC) for all obtained merged images using ImageJ software.^54^ For SC5C_60_serTRIAZOLE, lysosomal colocalization was characterized by very high PCCs and MOCs (above 0.83, Table S3). On the other hand, both coefficients indicated a low affinity of SC5C_60_serTRIAZOLE toward mitochondria (PCC = 0.55 and MOC = 0.31). For the second triazole, the calculated correlation coefficients were above 0.74 for lysosomes and in the range of 0.5–0.67 for mitochondria. Additionally, control experiments that stained with SC5 dye alone showed that the dye had no affinity toward lysosomes (Figure S20). In this case, PCC and MOC were 0.413 and 0.293, respectively.

## Conclusions

In summary, we synthesized triple-bonded symmetrical (*T*_*h*_) fullerene hexakisadduct TBC_60_ser, which was characterized spectrally (NMR, FT-IR, XPS measurements), followed by mass spectrometry and TEM/DLS studies. The ^13^C NMR spectra confirmed its high symmetry (two fullerene sp^2^ and one sp^3^ carbon), whereas triple bonds were confirmed by FT-IR and XPS. The obtained [60]fullerene nanomaterial was further used as a probe for cellular visualization of nonfluorescent buckyballs in a breast cancer model. Interestingly, the described protocol allowed the detection of [60]fullerene derivatives in the presence of FBS proteins. This observation is of practical importance due to the formation of protein coronas on the buckyball surface, which did not disturb the method’s efficacy. Interestingly, colocalization studies revealed that TBC_60_ser localized in lysosomes of the MCF-7 cells with a low affinity to mitochondria. Further studies should be performed for finding appropriate azidofullerenes that are water-soluble and penetrate cell membranes, which could be used as a partner for strain-promoted click reactions in animals.

## Supporting Information Available

Synthetic procedures; NMR and FT-IR spectroscopies; mass spectrometry (MALDI, ESI); UV–vis spectroscopy; DLS and ζ measurements; XPS spectroscopy; biological properities of TBC_60_ser and fullerene triazoles (PDF)
