# Supplementary material for: In Situ Cellular Localization of Nonfluorescent [60]Fullerene Nanomaterial in MCF-7 Breast Cancer Cells
Source: ACS Biomater Sci Eng. 2022 Jul 20;8(8):3450–62. doi: 10.1021/acsbiomaterials.2c00542 (PMC9364322; doi:10.1021/acsbiomaterials.2c00542)
Supplement: Supplementary file 1 — ab2c00542_si_001.pdf [file ab2c00542_si_001.pdf]

In situ cellular localization of non-fluorescent [60]fullerene nanomaterial in a MCF-7 breast cancer cells

Maciej Serda[1], Katarzyna Malarz[2,3], Julia Korzuch[1], Magdalena Szubka[2,3], Maciej Zubko[4,5], and Robert Musioł[1]

[1]*Institute of Chemistry, University of Silesia in Katowice, Katowice, 40-006, Poland*

[2]*Silesian Center for Education and Interdisciplinary Research, 75 Pulku Piechoty 1a, 41-500 Chorzow, Poland*

[3]*Chełkowski Institute of Physics, University of Silesia in Katowice, 75 Pulku Piechoty 1, 41-500 Chorzow, Poland*

[4]*Institute of Materials Science, University of Silesia in Katowice, Chorzow, 75 Pulku Piechoty, 1A, 41-500, Poland*

[5]*Department of Physics, Faculty of Science, University of Hradec Králové, Rokitanského 62, 500 03 Hradec Králové, Czech Republic*

*corresponding author: maciej.serda@us.edu.pl

SYNTHETIC PROCEDURES

NMR AND FT-IR SPECTROSCOPIES

MASS SPECTROMETRY (MALDI, ESI)

UV-VIS SPECTROMETRY

DLS and ZETA MEASUREMENTS

XPS SPECTRPSCOPY

BIOLOGICAL PROPERITIES OF TBC$_{60}$ser AND FULLERENE TRIAZOLES

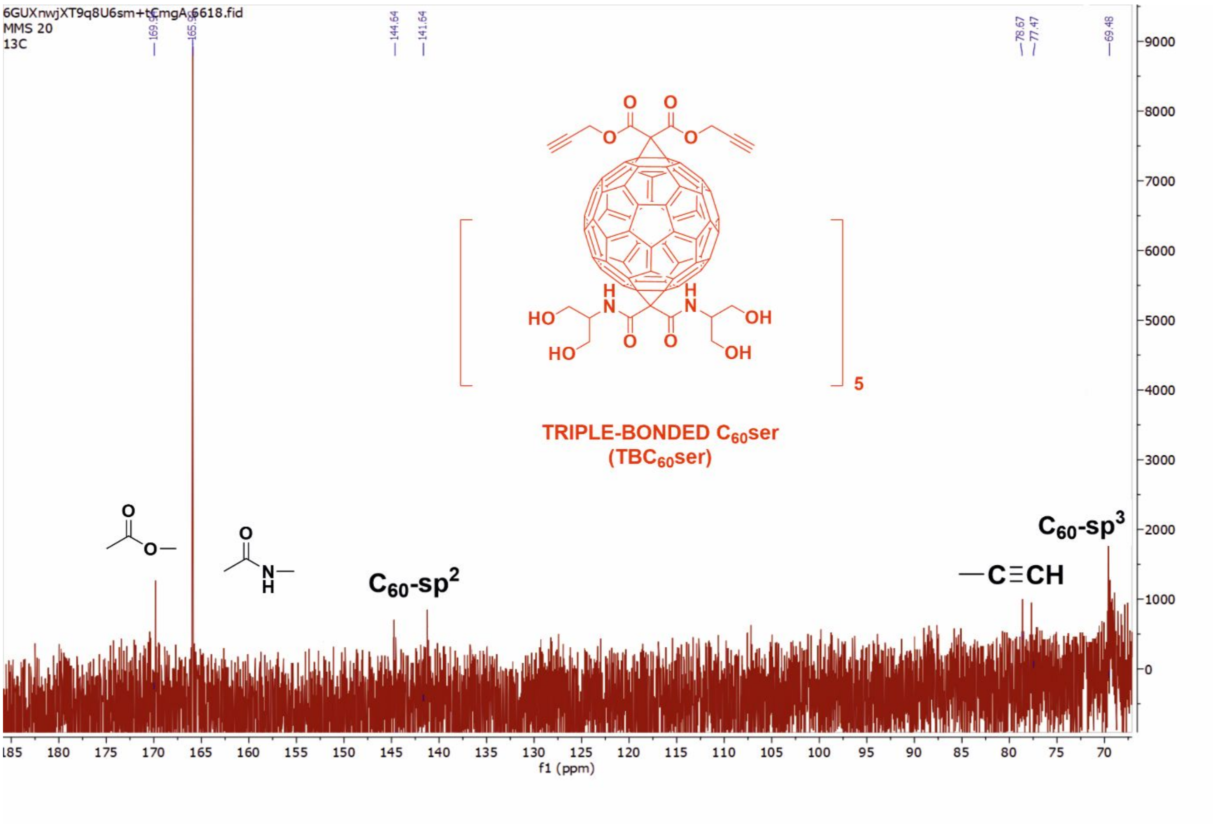

Figure S1

The fragment of ^{13}C-NMR of TBC$_{60}$ser confirming its T$_h$ symmetry.

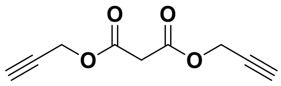

(1)

^{1}H-NMR(*500 MHz, CDCl$_3$, ppm*): 4.77(d, *J*= 2.5 Hz, 4H, -CH$_2$); 3.50 (s, 2H, O=C-CH$_2$-C-O), 2.53(m, 2H, C-H).

^{13}C-NMR(*125 MHz, CDCl$_3$, ppm*): 165.27; 76.79; 75.58; 53.04; 40.84.

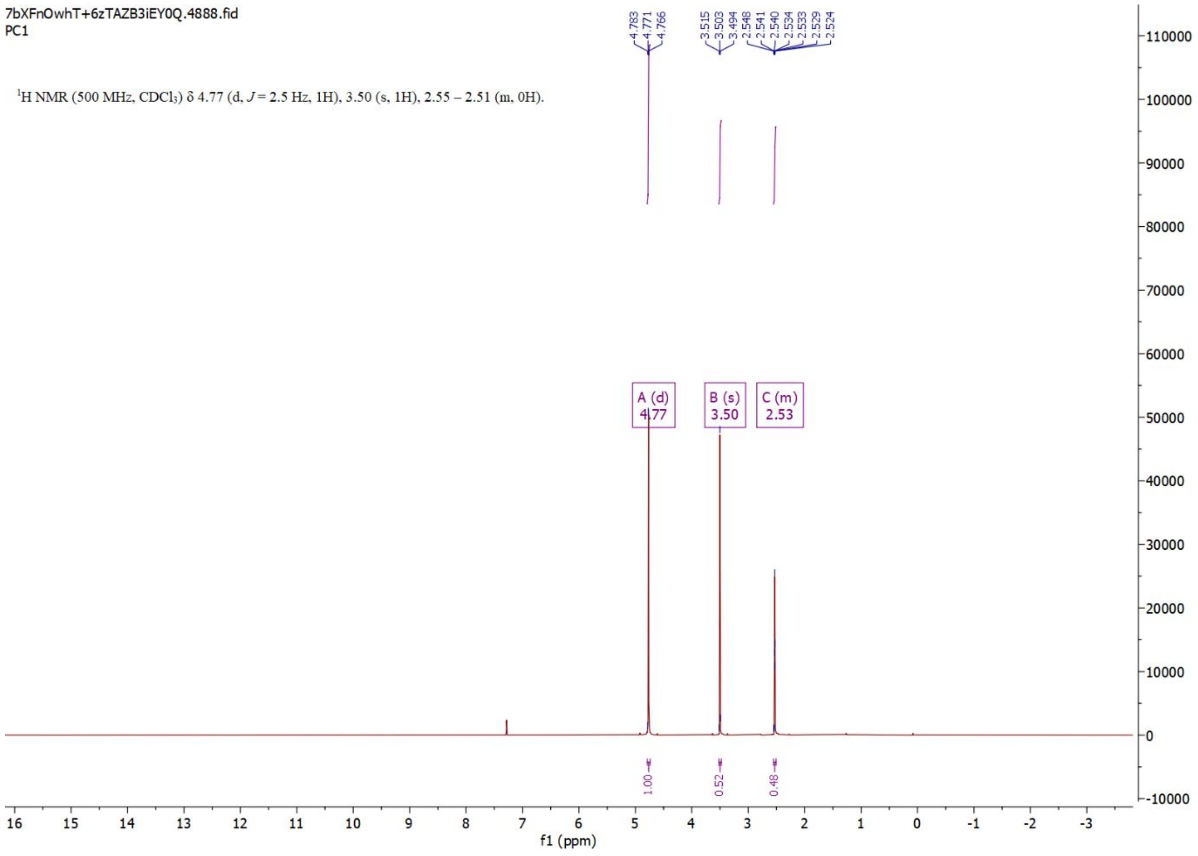

Figure S2

^{1}H-NMR of malonate (1) performed in CDCl$_3$.

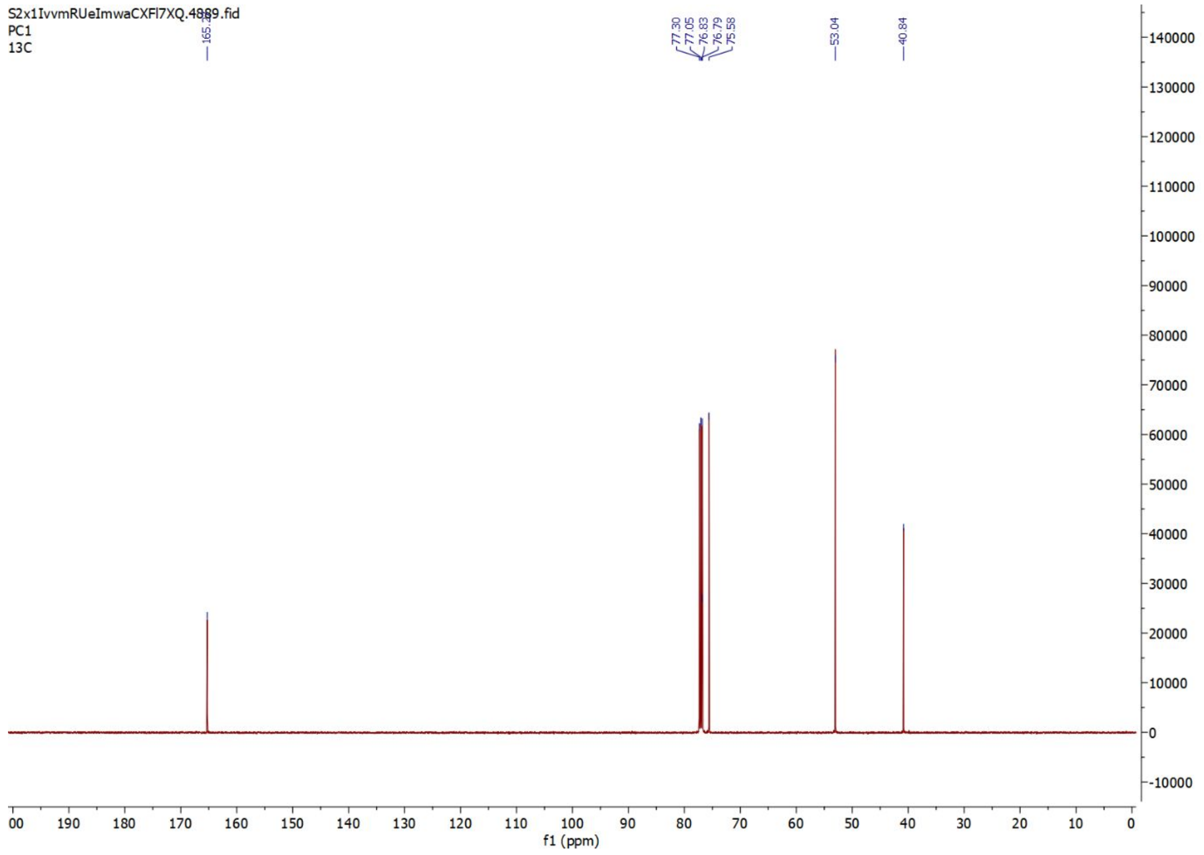

Figure S3

^{13}C-NMR of malonate (1) performed in $CDCl_3$.

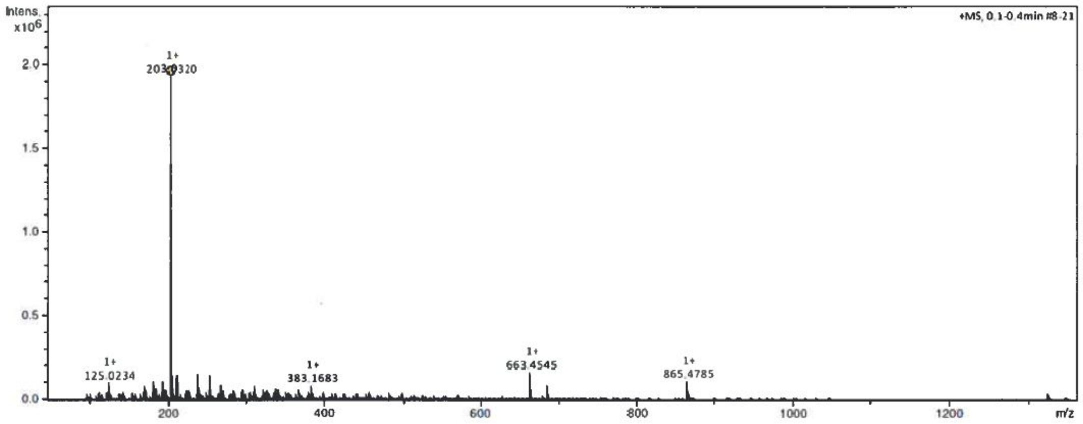

Meas. m/z	#	Ion Formula	m/z	err [ppm]	mSigma	# Sigma	Score	rdb	e— Conf	N-Rule
203.0320	1	**C9H8NaO4**	203.0315	**2.4**	14.5	1	100.00	5.5	even	ok

Figure S4

HRMS spectrum of malonate (1)

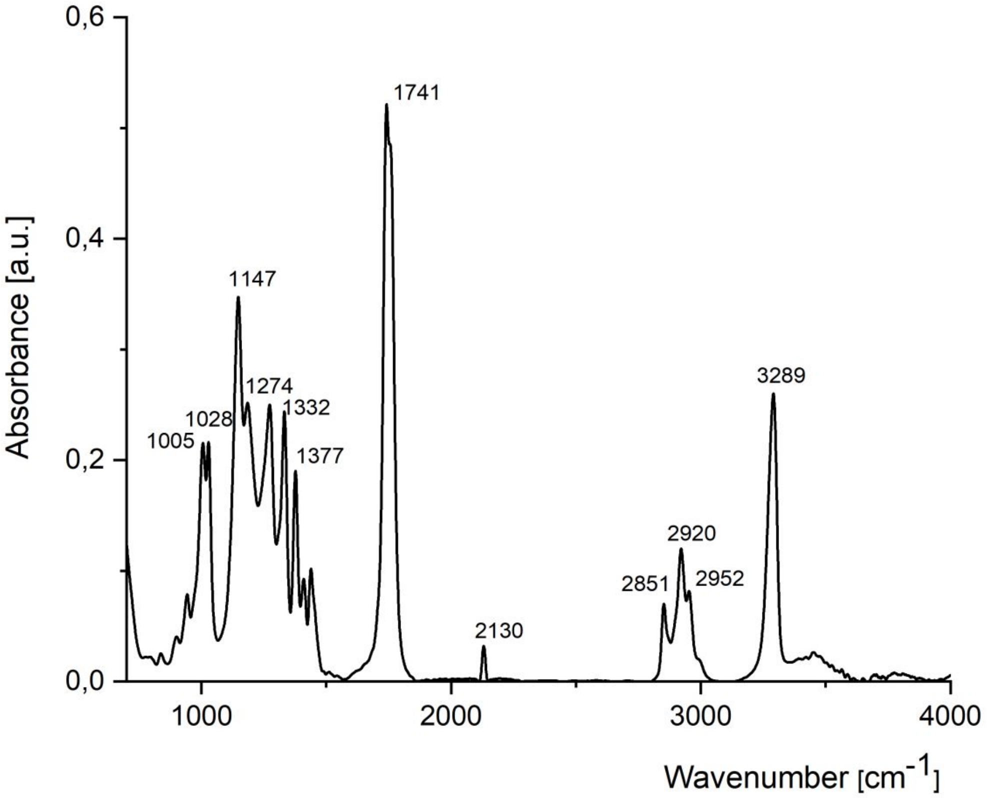

Figure S5

FTIR spectrum of malonate (**1**).

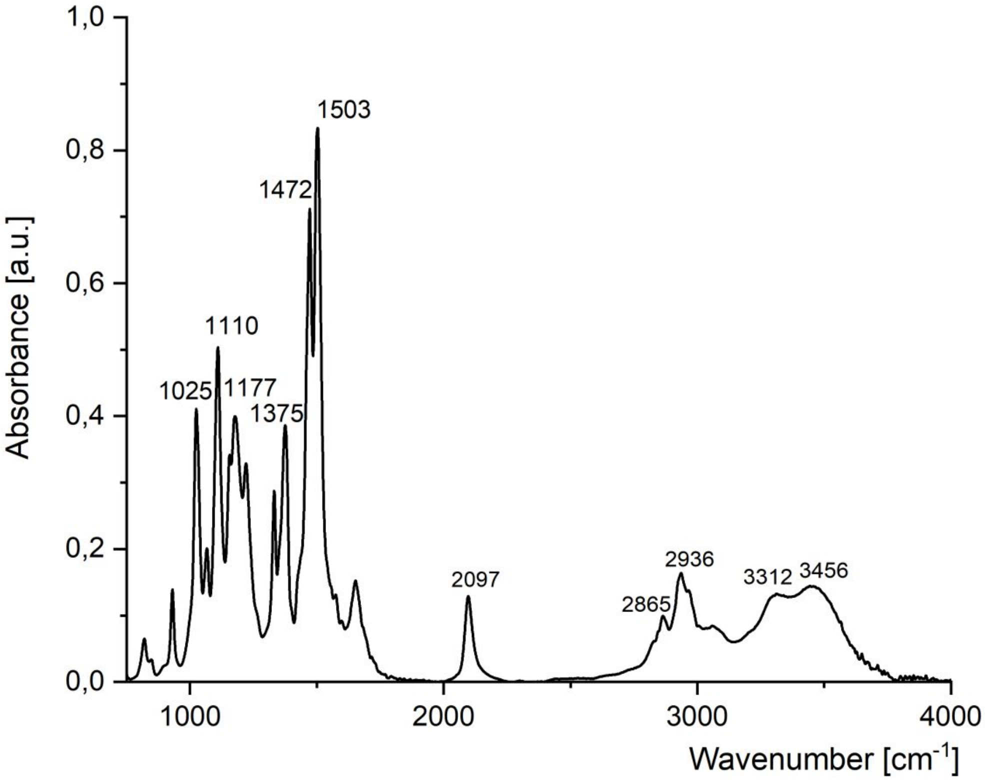

Figure S6

FTIR spectrum of sulfocyanine5 azide (SC5 azide)

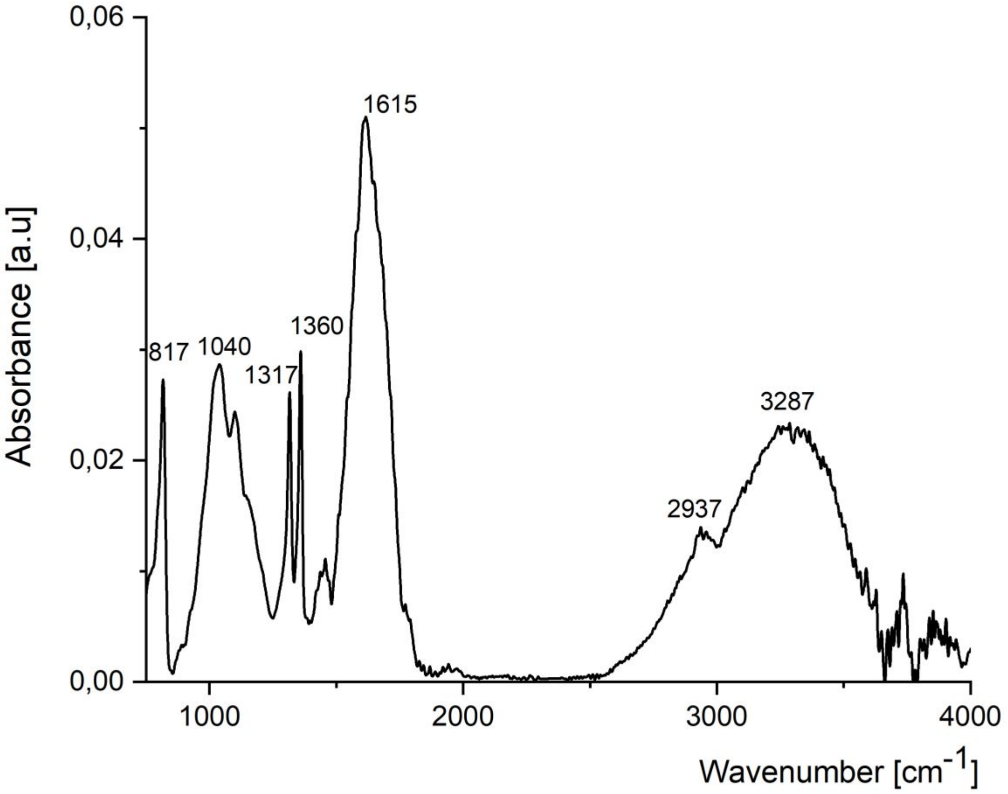

Figure S7

FTIR spectrum of fullerene triazole SC5C_{60}serTRIAZOLE

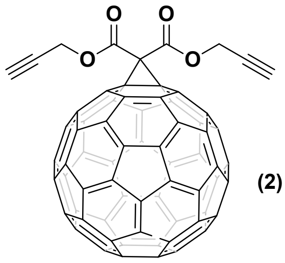

(2)

^{1}H-NMR(*500 MHz, $CDCl_3$, ppm*): 4.52 (m, 4H, -CH_2); 2.49(m, 2H, CH).

^{13}C-NMR(*125 MHz, $CDCl_3$, ppm*): 162.64 (C=O); 145.35 (C_{60}-sp^2); 145.25 (C_{60}-sp^2); 145.11(C_{60}-sp^2); 145.00(C_{60}-sp^2); 144.76(C_{60}-sp^2); 144.74(C_{60}-sp^2); 144.68(C_{60}-sp^2); 144.65(C_{60}-sp^2); 143.92(C_{60}-sp^2); 143.06(C_{60}-sp^2); 143.04(C_{60}-sp^2); 142.25(C_{60}-sp^2); 141.87(C_{60}-sp^2); 141.03(C_{60}-sp^2); 139.31(C_{60}-sp^2); 76.56 (C≡C); 75.87(C≡C); 70.66 (C_{60}-sp^3); 54.57(CH_2-O).

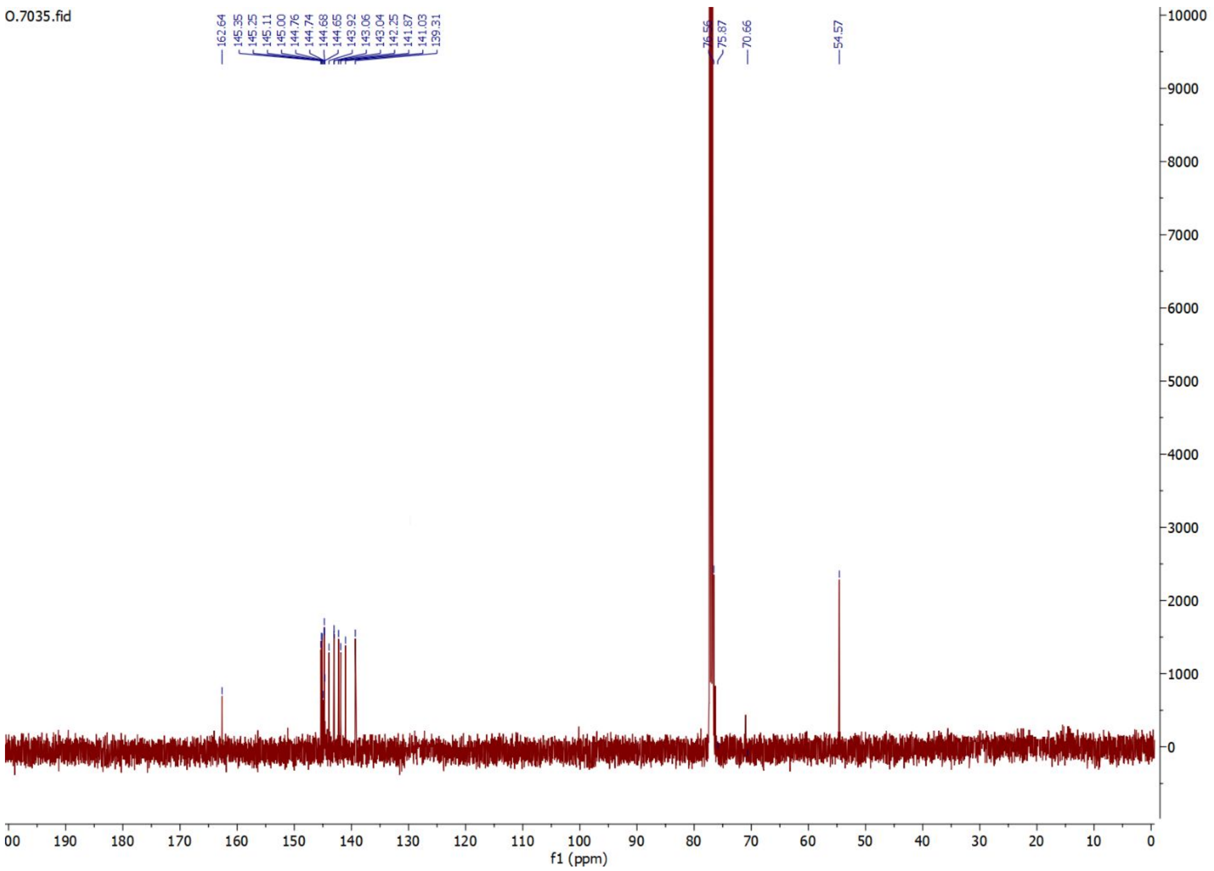

Figure S8

The ^{13}C-NMR of fullerene nanomaterial (**2**).

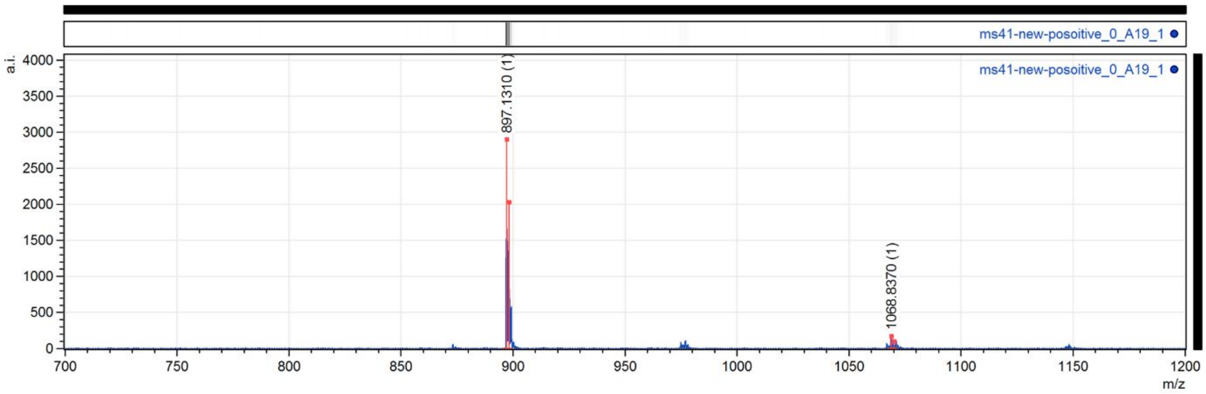

Figure S9

MALDI-TOF of fullerene nanomaterial (**2**)

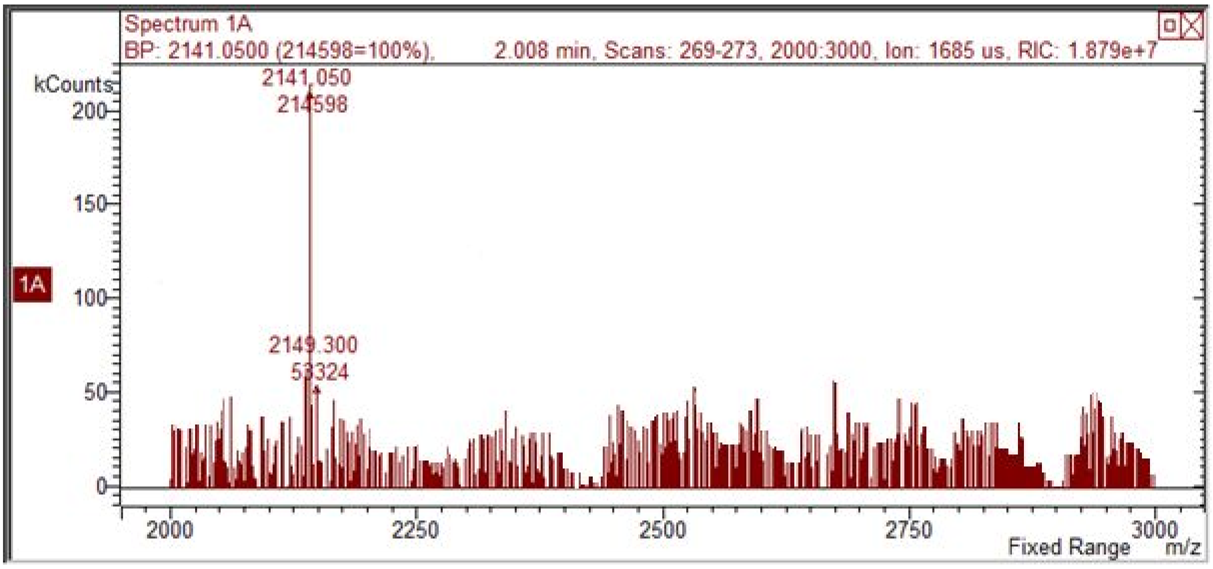

Figure S10

ESI-MS (positive mode) of fullerene nanomaterial (**4**) (+50 mV).

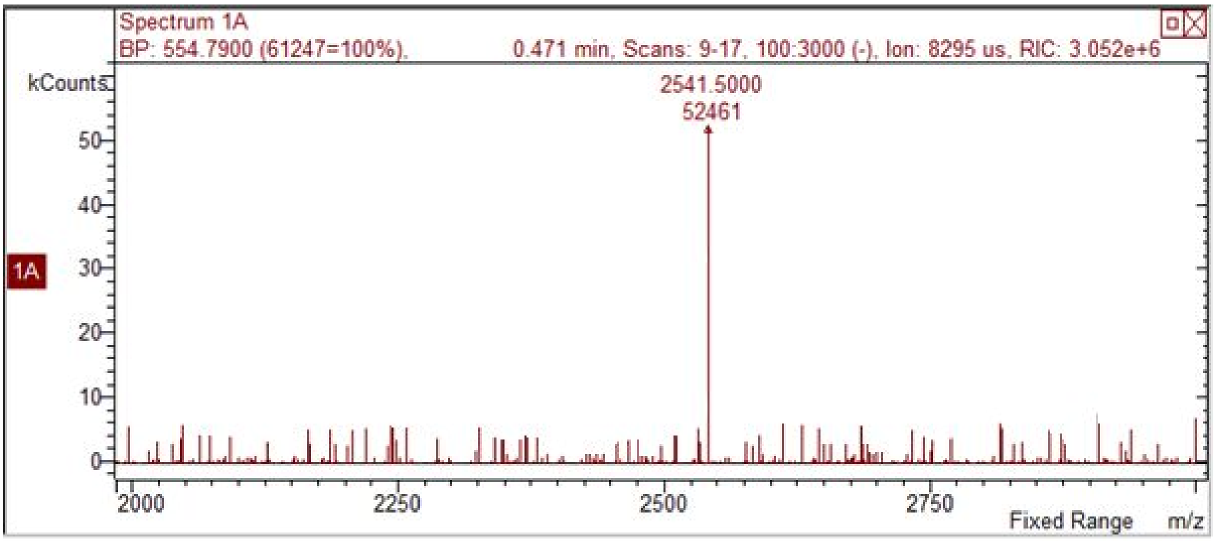

Figure S11

ESI-MS spectrum of (positive mode) of click reaction product HCC_{60}serTRIAZOLE (+50 mV).

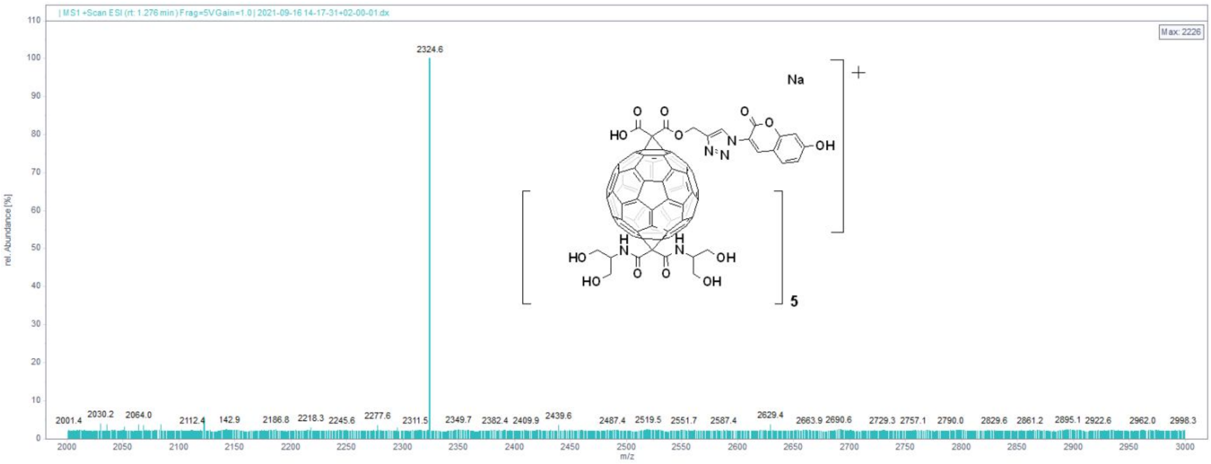

Figure S12

ESI-MS fragmentation spectrum (positive mode) of click reaction product HCC_{60}serTRIAZOLE (+300 mV).

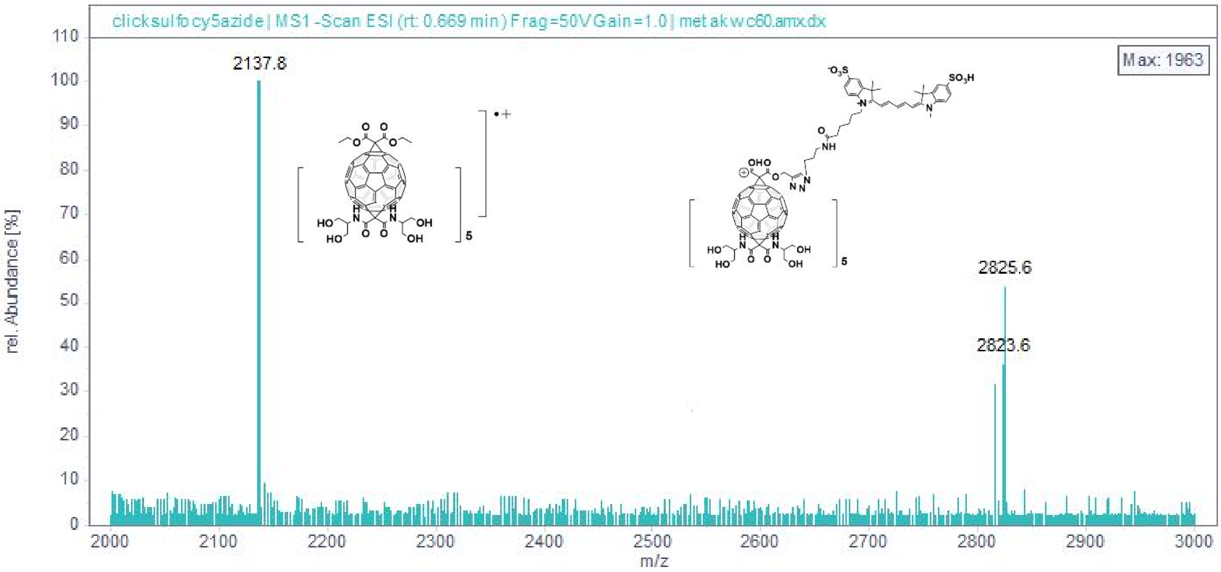

Figure S13

ESI-MS fragmentation spectrum (positive mode) of click reaction product SC5C_{60}serTRIAZOLE (+300 mV).

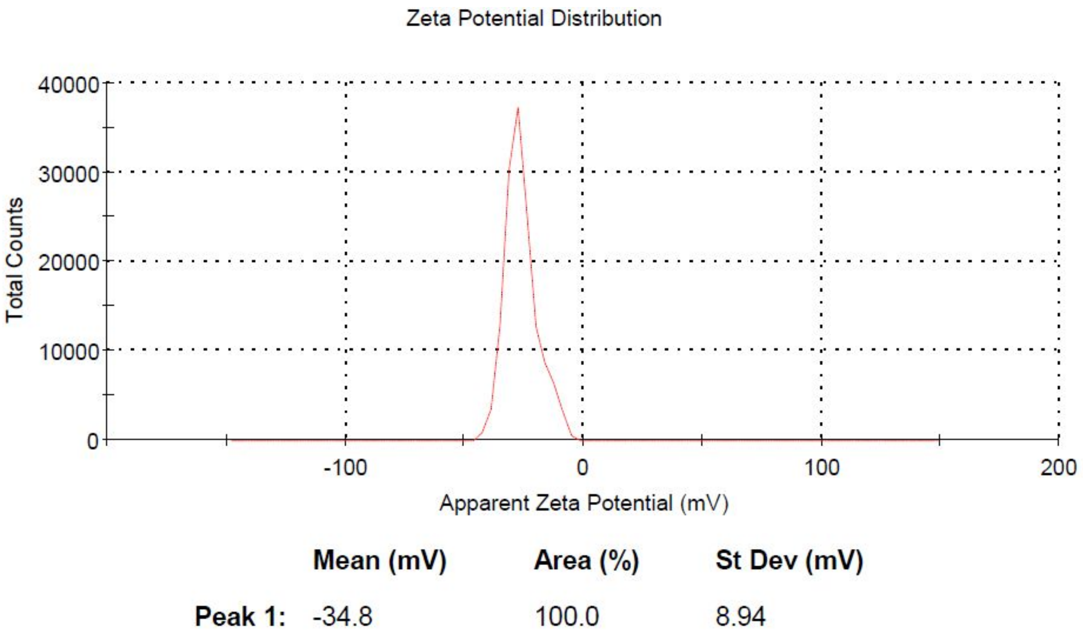

Figure S14

Zeta potential measurement of TBC_{60}ser in DI water (c=0.01 mg/ml)

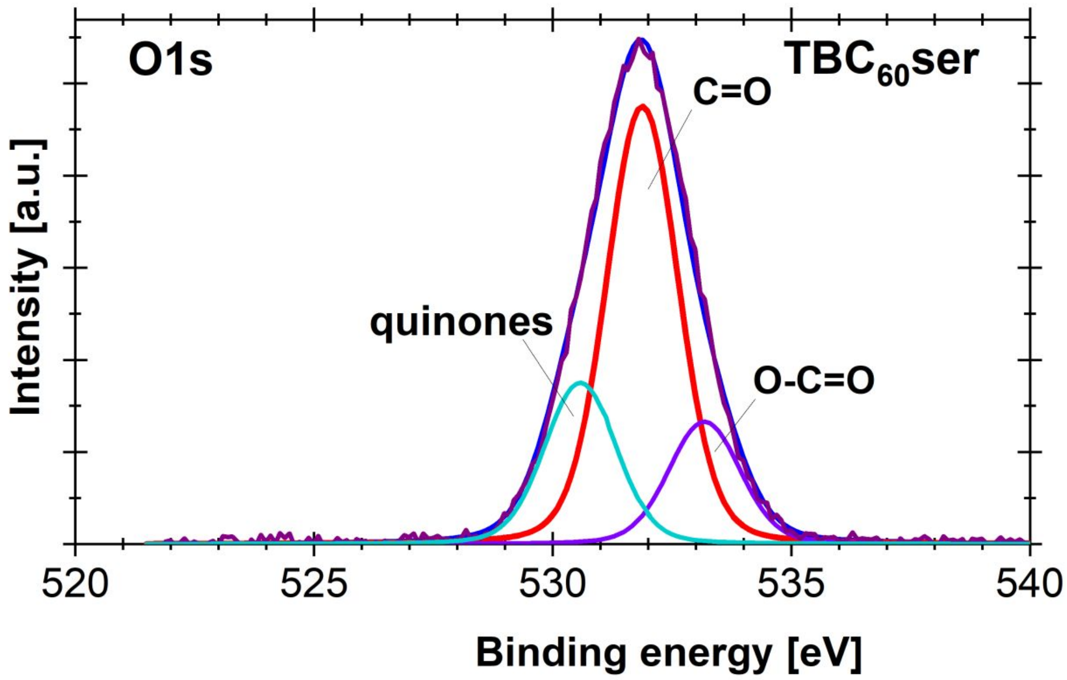

Figure S15

The XPS profile (O1s) of fullerene nanomaterial TBC_{60}ser.

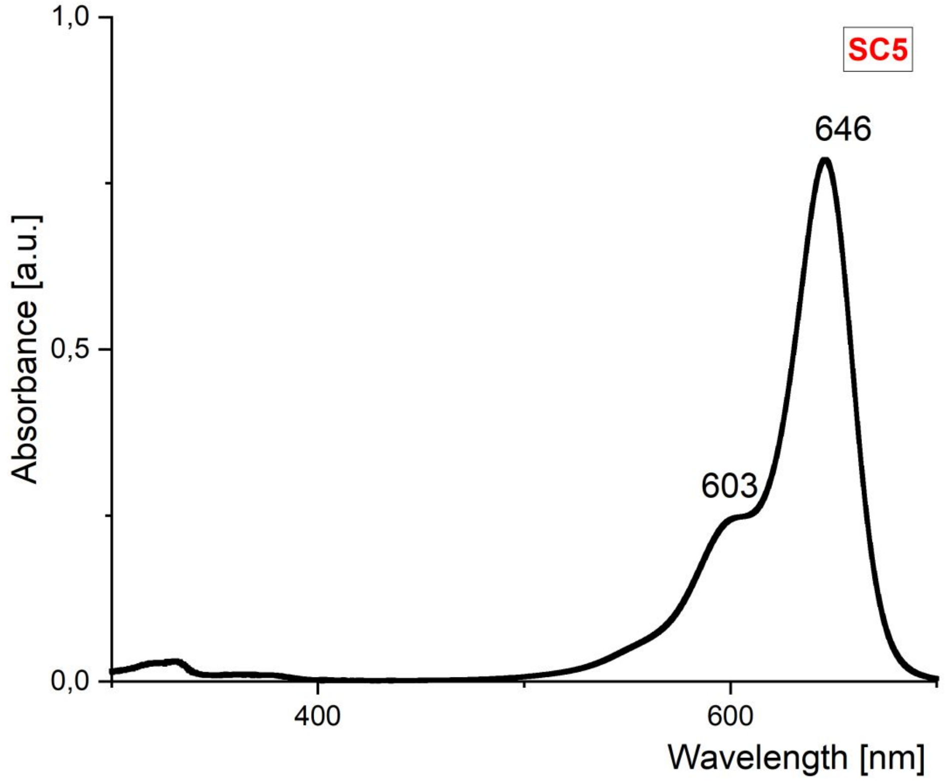

Figure S16

The UV-VIS spectrum of sulfo-cyanine5 azide (SC5) in water (c= 0.001 mg/mL).

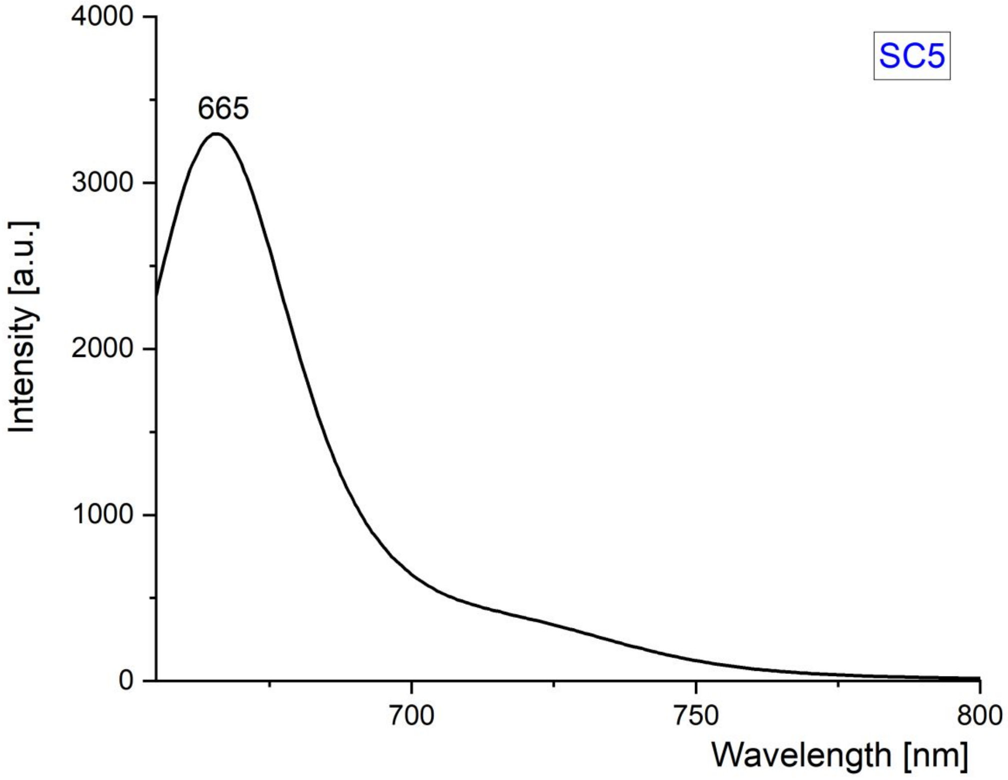

Figure S17

The fluorescence spectrum of sulfo-cyanine5 azide (SC5) in water (c= 0.001 mg/mL; ex= 646 nm).

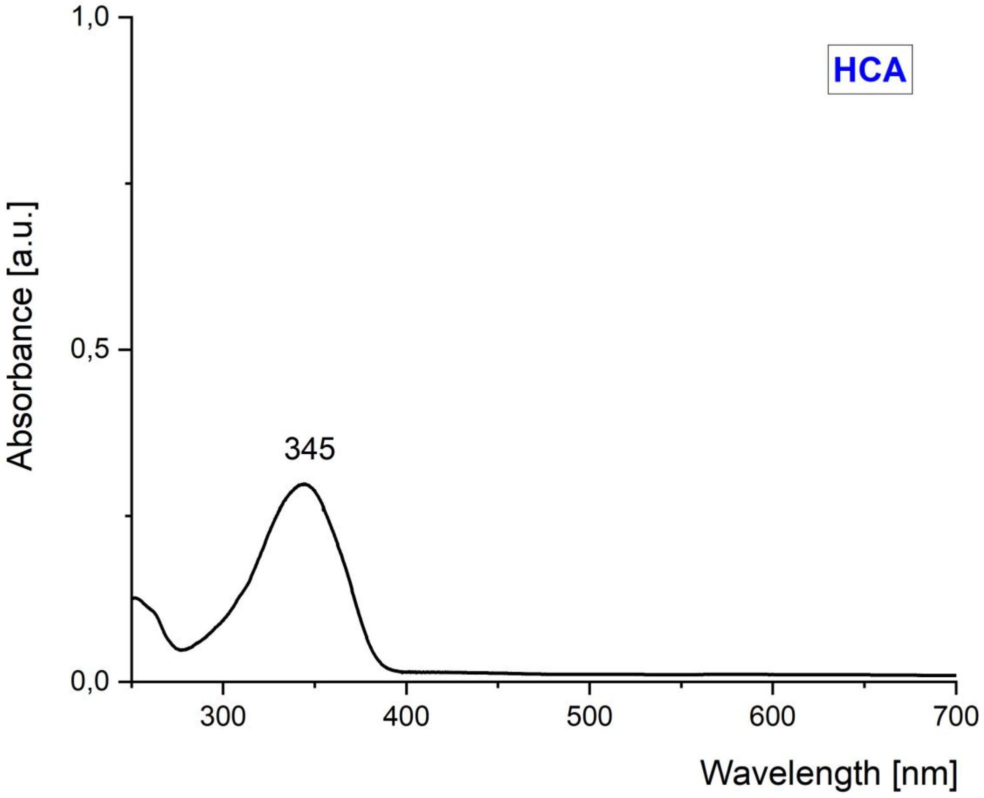

Figure S18

The UV-VIS spectrum of hydroxycoumarin azide (HCA) in ethanol (c= 0.0025 mg/mL).

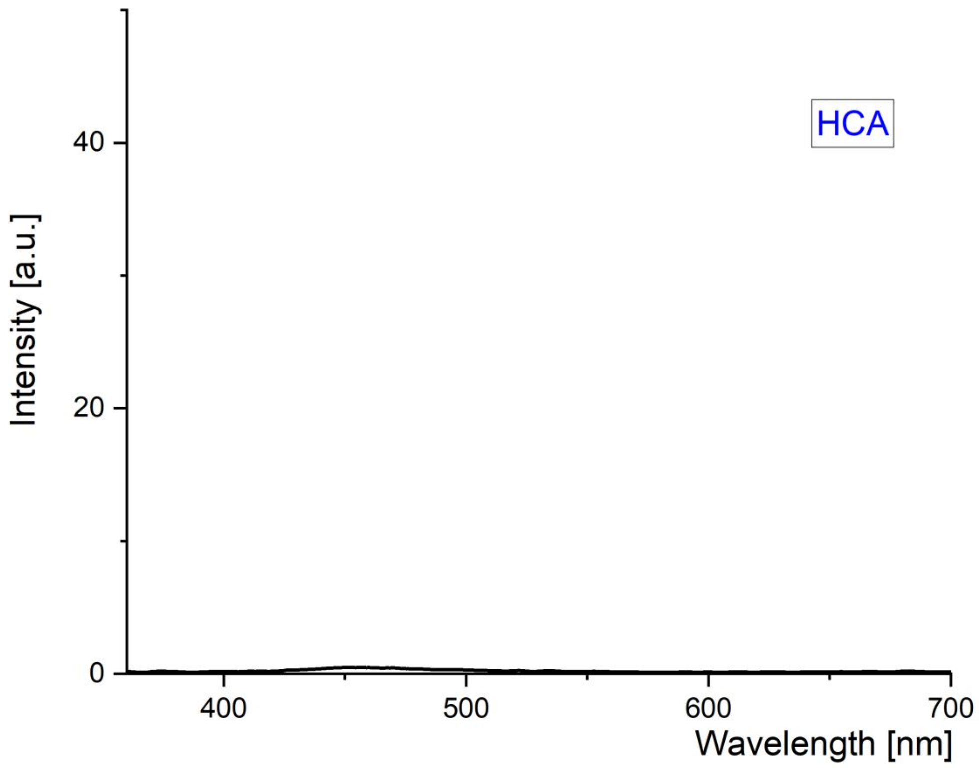

Figure S19

The fluorescence spectrum of hydroxycoumarin azide (HCA) in water (c= 0.0025 mg/mL; ex= 340).

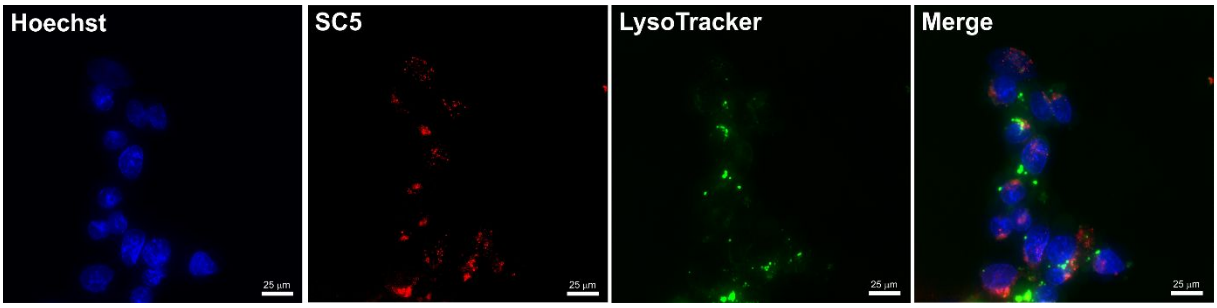

Figure S20

Colocalization image of SC5 azide (red) with lysotracker dye (green) in MCF-7 cells. Scale bars = 25 μm.

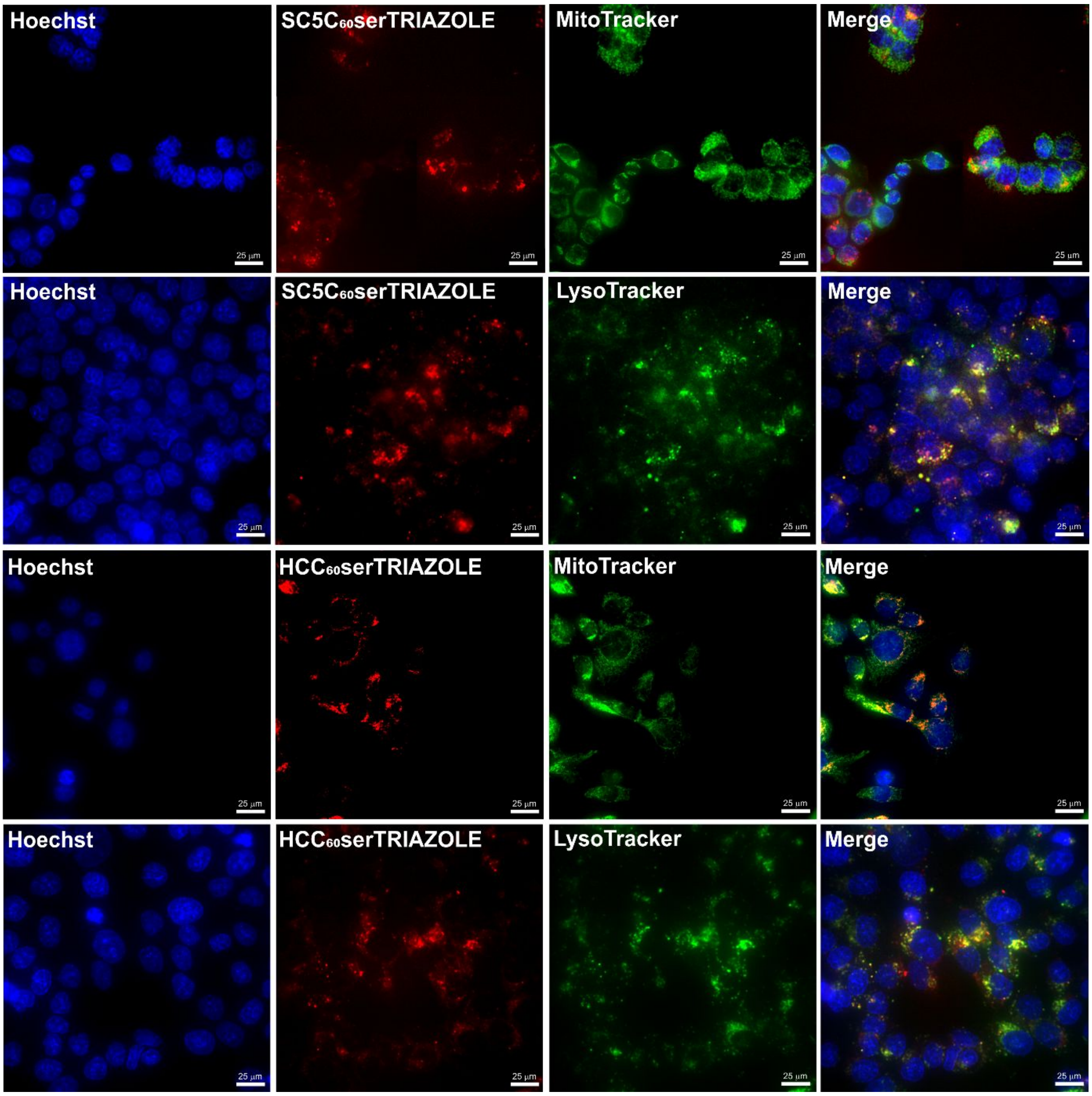

Figure S21

Cellular colocalization study of fullerene adducts: SC5C_{60}serTRIAZOLE and HCC_{60}serTRIAZOLE (red) with mitochondria/lysosomes (green). Cell nuclei are colored blue. Scale bars = 25 μm.

	$TBC_{60}ser$			
**Element**	**Atomic concentration [at %]**	**Chemical states**	**Binding energy [eV]**	**Contributions of lines [%]**
**C1s**	75.1	C≡C	283.1	3.8
		C-H, C-C	284.9	62.9
		C-O, C-N, -C-OH	286.5	25.3
		C=O	288.3	8.0
**O1s**	18.7	quinones	530.5	22.4
		C=O	531.9	60.7
		O-C=O	533.2	16.9
**N1s**	6.2	pyridinic N	398.3	37.3
		C-N, N-(C=O)-	399.8	62.7

Table S1

Chemical composition, atomic concentration and percentage contributions of chemical state for particular element in fullerene nanomaterial $TBC_{60}ser$

Component	Cytotoxicity (IC_{50})	
	MCF-7	NHDF
**TRIPLE-BONDED C_{60}ser (TBC_{60}ser)**	>468 µM (>1 mg/mL)	>468 µM (>1 mg/mL)
**SULFO-CYANINE5 AZIDE (SC5)**	>25 µM	>25 µM
**HYDROXYCOUMARIN AZIDE (HCA)**	>25 µM	>25 µM
**$CuSO_4$**	372 ± 18 µM	-

Table S2

Cytotoxicity of tested fullerene nanomaterial, azides and copper (II) sulfate against breast cancer cell line (MCF-7) and normal human fibroblasts (NHDF).

Component	**Mitochondria**		**Lysosome**	
	Pearson's	Manders'	Pearson's	Manders'
**$SC5C_{60}$serTRIAZOLE**	0.557	0.309	0.863	0.837
**HCC_{60}serTRIAZOLE**	0.670	0.505	0.787	0.741
**SC5**	-	-	0.413	0.293

Table S3

Correlation coefficients of colocalization images.
